# Depressive symptoms and neuroticism-related traits are the main factors associated with wellbeing independent of the history of lifetime depression in the UK Biobank

**DOI:** 10.1017/S003329172100502X

**Published:** 2023-05

**Authors:** Chiara Fabbri, Julian Mutz, Cathryn M. Lewis, Alessandro Serretti

**Affiliations:** 1 Department of Biomedical and Neuromotor Sciences, University of Bologna, Bologna, Italy; 2 Social, Genetic and Developmental Psychiatry Centre, Institute of Psychiatry, Psychology & Neuroscience, King's College London, London, UK

**Keywords:** Loneliness, major depressive disorder, neuroticism, wellbeing, polygenic risk score

## Abstract

**Background:**

Wellbeing has a fundamental role in determining life expectancy and major depressive disorder (MDD) is one of the main modulating factors of wellbeing. This study evaluated the modulators of wellbeing in individuals with lifetime recurrent MDD (RMDD), single-episode MDD (SMDD) and no MDD in the UK Biobank.

**Methods:**

Scores of happiness, meaningful life and satisfaction about functioning were condensed in a functioning-wellbeing score (FWS). We evaluated depression and anxiety characteristics, neuroticism-related traits, physical diseases, lifestyle and polygenic risk scores (PRSs) of psychiatric disorders. Other than individual predictors, we estimated the cumulative contribution to FWS of each group of predictors. We tested the indirect role of neuroticism on FWS through the modulation of depression manifestations using a mediation analysis.

**Results:**

We identified 47 966, 21 117 and 207 423 individuals with lifetime RMDD, SMDD and no MDD, respectively. Depression symptoms and personality showed the largest impact on FWS (variance explained ~20%), particularly self-harm, worthlessness feelings during the worst depression, chronic depression, loneliness and neuroticism. Personality played a stronger role in SMDD. Anxiety characteristics showed a higher effect in SMDD and no MDD groups. Neuroticism played indirect effects through specific depressive symptoms that modulated FWS. Physical diseases and lifestyle explained only 4–5% of FWS variance. The PRS of MDD showed the largest effect on FWS compared to other PRSs.

**Conclusions:**

This was the first study to comprehensively evaluate the predictors of wellbeing in relation to the history of MDD. The identified variables are important to identify individuals at risk and promote wellbeing.

## Introduction

Wellbeing is a complex concept that includes physical, mental and social components, which are associated with life expectancy (Ni et al., 2020). Previous studies have demonstrated that the psychosocial component is highly relevant and able to influence the physical component by modulating the risk of a number of diseases such as cardiovascular problems, cancer and Alzheimer's disease (Rico-Uribe et al., 2018).

In studies conducted in the general population, the most common way to measure wellbeing is through life evaluations, using life satisfaction questions and questions asking how happy people are with their lives (Helliwell, 2019). These studies have identified a number of variables associated with wellbeing, in particular social support, personality characteristics, chronic diseases such as psychiatric disorders and obesity, lifestyle and socioeconomic status (Helliwell, 2019; Santini et al., 2020; Spittlehouse, Vierck, Pearson, & Joyce, 2014; Stranges, Samaraweera, Taggart, Kandala, & Stewart-Brown, 2014).

Psychiatric disorders such as depression and anxiety are associated with reduced wellbeing (Santini et al., 2020). In major depressive disorder (MDD), most previous studies assessed how clinical characteristics of the disease affect wellbeing. Among symptoms reported during the depressive episode, difficulty concentrating and making plans were found to have the largest impact on psychosocial functioning, not only during the depressive phase but also after remission of other symptoms (Christensen, Wong, & Baune, 2020; McIntyre et al., 2013). Low self-esteem and lack of energy followed those symptoms in terms of impact on psychosocial functioning persisting after the acute phase (Christensen et al., 2020). Several clinical features were associated with worse prognosis of MDD, including the duration of the depressive episode and total duration of the illness, suicidality, anxiety and physical diseases such as cardiovascular disease and diabetes (Kraus, Kadriu, Lanzenberger, Zarate, & Kasper, 2019). These factors were associated with poorer response to treatments and persistence of depressive symptoms; lack of symptom remission has been strongly associated with poorer quality of life (IsHak et al., 2015). However, as previously noted, some symptoms may persist even when the overall depression is remitted and they are associated with persistent functional impairment. Therefore, an individual's wellbeing should be considered as a key outcome in patients with MDD, not only symptom remission (IsHak et al., 2011).

A systematic evaluation of the variables modulating wellbeing in individuals with lifetime MDD is still lacking, as well as a comparison with those playing a role in the population without history of lifetime MDD. Another stratification factor of particular interest is recurrence of MDD. This has been associated with markers of severity, such as longer depressive episodes, melancholia, suicidal ideation, higher number of depressive symptoms, psychiatric comorbidities such as anxiety and substance use disorders, higher neuroticism, cognitive symptoms and functional impairment (Burcusa & Iacono, 2007; Wakefield & Schmitz, 2013). However, the possible relevance of specific variables in modulating the wellbeing of this group compared to those with single-episode MDD (SMDD) is unclear.

Other than being influenced by environmental factors, wellbeing has a significant genetic component (heritability from 23 to 47%) and high negative genetic correlations (⩾−80%) with neuroticism and depressive symptoms (Baselmans et al., 2019; Jamshidi et al., 2020; Okbay et al., 2016). No significant genetic correlation of wellbeing with physical health phenotypes was found, for example, with body mass index and coronary artery disease (Okbay et al., 2016). The genetic correlation of depression with physical health traits was instead shown to be statistically significant (Howard et al., 2019), suggesting specific genetic factors that diverge between depression and wellbeing.

Despite the clinical and genetic evidence of associations between psychiatric disorders and wellbeing, the role of polygenic risk scores (PRSs) of major psychiatric disorders in predicting wellbeing has not been systematically explored in previous studies, to the best of our knowledge.

The importance of wellbeing in determining health and life expectancy drives the need for a better understanding of the variables that have a relevant impact on it. This study provides a comprehensive assessment of the effect of different types of variables on wellbeing in the UK Biobank (UKB). Analyses were stratified by history of lifetime recurrent MDD (RMDD), lifetime SMDD and no lifetime MDD, as we hypothesized possible differences among these groups, which may be relevant for disease prognosis and/or for defining the risk of poor health outcomes.

## Methods

### Sample and MDD definition

UKB is a prospective population-based study of ~500 000 individuals recruited from across the UK except Northern Ireland, aged between 37 and 73 at baseline. Further information on UKB is included in the online Supplementary Methods.

Lifetime MDD was defined as satisfying at least one of the following: (1) at least five symptoms of depression assessed by the Composite International Diagnostic Interview Short Form (CIDI-SF) (Davis et al., 2020); (2) Smith et al. criteria (Smith et al., 2013); (3) ICD-10 codes for MDD (F32-F33), considering both primary and secondary diagnoses; (4) at least one diagnostic code for a unipolar depressive disorder in primary care records (Fabbri et al., 2021). Minimal phenotyping of MDD in UKB was reported to select individuals with different characteristics compared to more strict phenotyping (Cai et al., 2020); therefore, measures of depression based on a single self-reported item were not considered (self-reported depression and help-seeking anxiety-depression, data fields 20 002, 2090 and 2100).

RMDD was determined as one or more of the following: (1) more than one depressive episode according to the Mental Health Questionnaire (MHQ) (data fields 20 442, 4620, 20 124, 20 125); (2) reported age at first episode of depression (data field 20 433) lower than age at last episode of depression (data field 20 434); (3) at least two diagnostic records of depression in different years in primary care data.

Participants with psychotic, bipolar and substance use disorders [according to the MHQ (Davis et al., 2020), ICD-10 codes and primary care records] were excluded from both the group with lifetime MDD and that without lifetime MDD, as these disorders have been associated with different clinical course and higher functional impairment compared to MDD (Davis et al., 2005; Sanchez-Moreno et al., 2009; Velthorst et al., 2017).

Genome-wide genetic data have been collected on all UKB participants (Bycroft et al., 2018).

### Measurement of functioning and wellbeing

We considered items in the following four categories:

1. Happiness (data fields 20 458 and 4526);
2. Belief that own life is meaningful and worth living (data fields 20 460 and 20 479);
3. Happiness with own health (data fields 20 459 and 4548);
4. Satisfaction about functioning in relevant areas of life (data fields 4559, 4570 and 4537 for family, friendship and work satisfaction, respectively).

We did not include financial situation satisfaction (data field 4581), because it reflects more on an individual's economic condition rather than the perceived happiness or satisfaction in one's own life and personal achievements. We adjusted analyses for the Townsend deprivation index to avoid capturing differences related to socio-economic status (SES, see Statistical analysis).

When multiple time points were available (e.g. happiness, data field 4526, had four instances that span different years), we calculated the average across the different instances, to provide a measure that was more robust to fluctuations due to changes in mood/depressive symptoms and/or transient external circumstances (the scores at different time points are in online Supplementary Fig. S1). We harmonized the coding of all items to make higher scores correspond to better wellbeing/functioning. Then we calculated the average score in each category and rescaled items in category (2) to 1–6 to make them comparable to items in the other categories. Finally, in participants having non-missing values for at least two of the four categories (‘I don't know’ and ‘prefer not to answer’ were considered as missing), we created a single score representing their average and we defined this as the functioning-wellbeing score or FWS (range 1–6). We calculated Pearson's correlation between the FWS and the score in each of the four categories to determine how much the FWS captured each area of wellbeing-functioning. We compared the FWS with the score obtained using a factor analysis, showing a strong correlation of 0.92 (details in the online Supplementary Methods and Supplementary Fig. S2).

Some of the measures included in the FWS were part of the MHQ (data fields 20 458, 20 460, 20 479 and 20 459) which was also used to define lifetime MDD according to the CIDI-SF. We excluded those with current MDD at the time of the MHQ [five or more depressive symptoms for more than half the days during the last 2 weeks, according to the self-report Patient Heath Questionnaire (PHQ)-9, *n* = 2507 (Löwe, Unützer, Callahan, Perkins, & Kroenke, 2004)], to limit the effect of acute depressive symptoms on items in the FWS. Other data fields included in the FWS were part of the psychosocial factors evaluated through the touchscreen questionnaire at assessment centres, but we could not assess if participants may have had a MDD episode at that time (only three data fields about depressed mood/unenthusiasm/tiredness in the last 2 weeks were available).

### Examined predictors of functioning and wellbeing

We selected variables pertaining to the following groups: (1) depressive symptoms and characteristics of periods of depression; (2) anxiety symptoms and characteristics of periods of anxiety; (3) neuroticism-related personality traits; (4) physical diseases and lifestyle; (5) PRSs of psychiatric disorders [MDD, schizophrenia, bipolar disorder, attention-deficit hyperactivity disorder (ADHD), alcohol dependence, anxiety disorders] and neuroticism. These were selected because they represent domains or genetic factors previously associated with the course, functional impact or treatment outcomes of MDD and/or wellbeing in the general population (Bartova et al., 2019; Cho et al., 2019; Christensen et al., 2020; Fabbri et al., 2021; Fanelli et al., 2021; Helliwell, 2019; McIntyre et al., 2013; Pain et al., in press; Santini et al., 2020; Spittlehouse et al., 2014; Stranges et al., 2014). For the selection of PRSs to include in the study, we also considered the availability of summary statistics from genome-wide association studies without sample overlap with UKB, to avoid overfitting (Choi, Mak, & O'Reilly, 2020). A description of each variable is provided in online Supplementary Table S1. The selected variables were usually available only in subsets of the sample, as reported in the Results paragraph.

### Statistical analysis

We evaluated the impact of each variable on FWS in individuals with RMDD, SMDD and no history of MDD separately, using linear regression models adjusted for age and Townsend deprivation index (as a proxy measure of SES). In order to make effect sizes comparable across different variables and between continuous and binary variables, we scaled continuous variables by subtracting the mean and dividing by two times the standard deviation, while dichotomous variables were centred by subtracting the mean (Gelman, 2008).

PRSs were created using PRS-CS-auto, a Bayesian method to estimate variants weights that do not need *a priori* definition of a *p*-threshold for selecting variants to include in the PRS (Ge, Chen, Ni, Feng, & Smoller, 2019); the genome-wide summary statistics are listed in online Supplementary Table S1. Non-strand ambiguous SNPs available in HapMap3 data were selected from the base summary statistics and imputed genome-wide data in UKB (details are reported in online Supplementary Methods). After the estimation of variant weights using PRS-CS-auto, we calculated PRSs by the score function in PLINK 2.0 (Chang et al., 2015). PRSs were scaled as described above and their association with FWS was investigated in each group of individuals using linear regression models adjusted for age, Townsend deprivation index, the first six population principal components [demonstrated to be ancestry-informative (Coleman et al., 2020)], assessment centre and genotyping batch. The power of PRS analyses was estimated using the R library ‘avengeme’.

Then, for each of the predictor categories, we evaluated the combined effect of statistically significant variables on the FWS by using elastic net regression with the previously described covariates. We used the R package ‘ensr’ to select the *α* value (shrinkage parameter) showing the minimum cross-validation error using 10-fold cross-validation and then the *R* package ‘glmnet’ to tune *λ* (penalty parameter) with 10-fold cross-validation to obtain the minimum mean-squared error. The variance explained by each model was calculated (*R*^2^).

Given the high correlation between neuroticism, depressive symptoms and wellbeing in the previous literature, we also tested the hypothesis of indirect effects of neuroticism on FWS through the modulation of depressive symptoms. We assumed that personality traits would be stable during an individual's lifetime, limiting the potential bias deriving from the use of cross-sectional data, though our approach cannot conclude the presence of a causal link between neuroticism and wellbeing. We used the *R* package ‘mediation’ to disentangle the direct and indirect (through the modulation of depressive symptoms) effects of neuroticism on the FWS. Depressive symptoms/characteristics having a statistically significant effect on FWS in at least two groups of individuals were considered. The same covariates previously mentioned were used. Finally, as a further exploration of the connection between depressive symptoms and neuroticism, we also tested the additional variance explained by adding neuroticism to depressive symptoms/characteristics.

Analyses were adjusted for multiple testing using the Bonferroni correction (see details about the *α* level in the result tables).

## Results

We identified 47 966, 21 117 and 207 423 individuals with lifetime RMDD, SMDD and no MDD, respectively, having at least two measures included in the FWS (see online Supplementary Table S2 for the clinical-demographic features and online Supplementary Fig. S3 for the distribution of the FWS in each group). The distribution of the different measures of MDD and their overlap is shown in online Supplementary Table S3; as expected, the overlap was lower for SMDD than for RMDD.

**Fig. 1.**
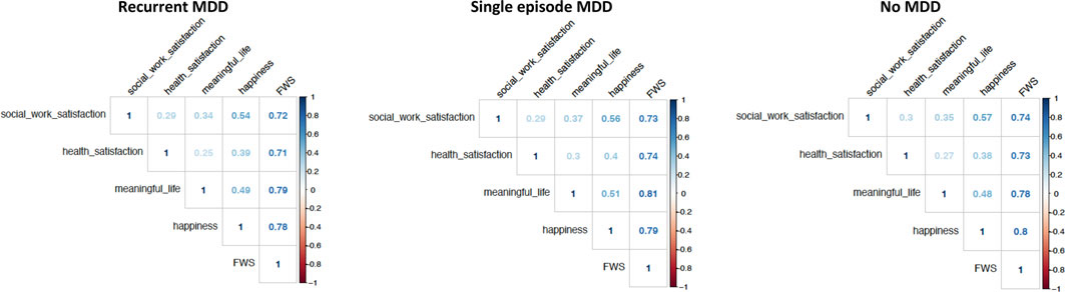
Pearson's correlation coefficients between each of the four functioning-wellbeing components and FWS (functioning-wellbeing) in participants with lifetime recurrent MDD, single-episode MDD and no MDD.

The scores in each of the four categories (happiness, life meaningfulness, health satisfaction, social/work satisfaction) were highly correlated with the FWS, suggesting that the latter captures the considered categories well, with a very similar pattern among RMDD, SMDD and no MDD; in the single categories, the highest correlation was found between happiness and social/work satisfaction (Fig. 1).

### Predictors of functioning/wellbeing

**Fig. 2.**
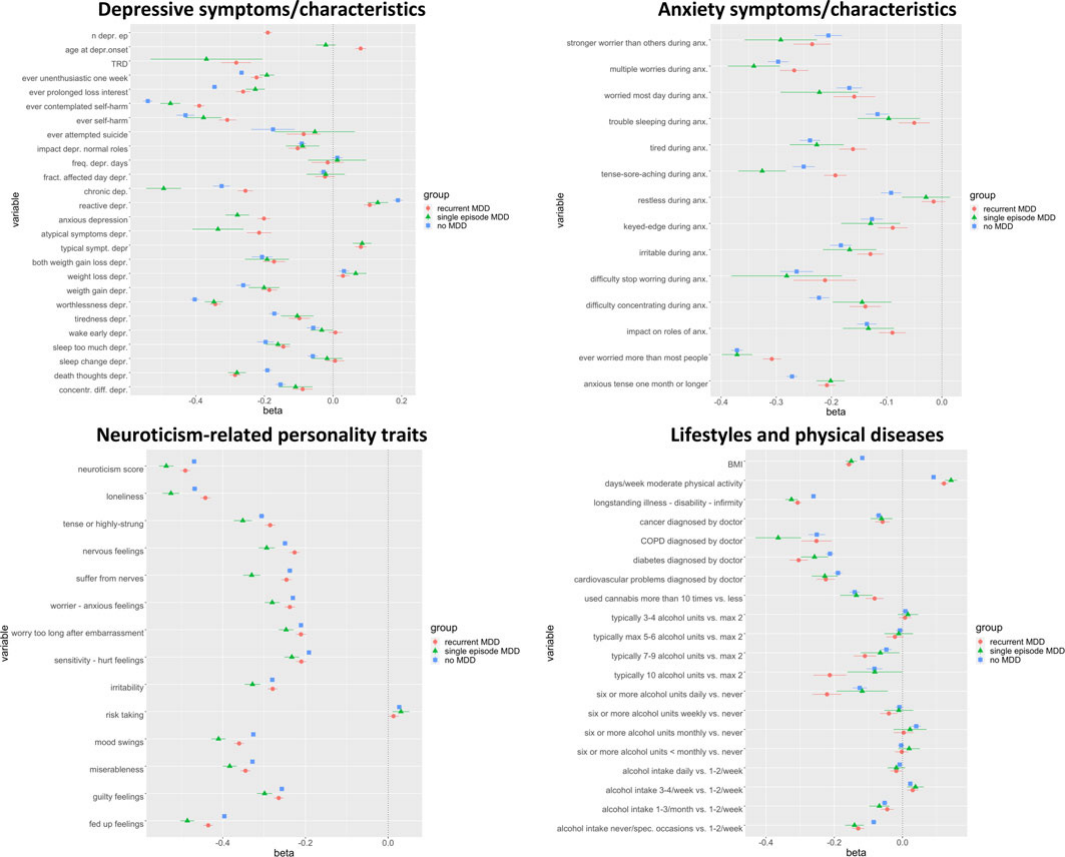
Effect size and 95% confidence intervals for the variables tested for association with functioning-wellbeing (FWS). MDD, major depressive disorder; TRD, treatment-resistant depression; Depr, depression; Anx., anxiety; COPD, chronic obstructive pulmonary disease; BMI, body mass index.

**Fig. 3.**
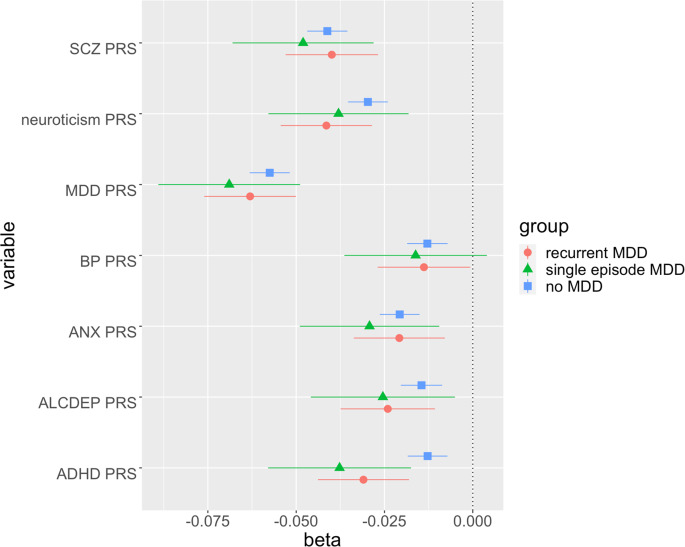
Effect size and 95% confidence intervals for the polygenic risk scores (PRS) tested for association with functioning-wellbeing (FWS). PRS were scaled to make the coefficients comparable with other tested variables (see Statistical analysis). SCZ, schizophrenia; MDD, major depressive disorder; BP, bipolar disorder; ANX, anxiety disorders; ALCDEP, alcohol dependence; ADHD, attention-deficit hyperactivity disorder.

Overall, the examined predictors had relevant differences in their effect size on FWS (Figs 2 and 3; online Supplementary Tables S4 and S5 show the distribution of the variables in each group and the results of the regression analyses, respectively). The number of individuals with non-missing data for the examined predictors was variable, as most predictors were not collected in the whole sample, and some questions were skipped based on previous answers (online Supplementary Table S4).

The largest effect size was found for the variables ever contemplated self-harm, ever self-harm, feelings of worthlessness during the worst depressive period, chronic depression, loneliness feelings, neuroticism and fed-up feelings (*β* ⩾ 0.40 in at least one group of individuals). Depression subtypes that were associated with lower FWS were anxious MDD and MDD with atypical neurovegetative symptoms, particularly in participants with SMDD compared to RMDD. On the other hand, MDD reactive to a stressful event was associated with higher FWS. Neuroticism-related personality traits were associated with poorer FWS, particularly in individuals with SMDD compared to the other groups. Among symptoms of anxiety, the variable ‘ever worried more than most people’ had the largest impact on FWS, in all groups.

Considering lifestyle and physical diseases, moderate physical activity had a positive impact on FWS, which was higher in individuals with MDD compared to those without lifetime MDD. Among physical diseases, longstanding illnesses, disabilities or infirmities, chronic obstructive pulmonary disease and diabetes had the largest effect on FWS in all groups. Alcohol use did not have a strong impact on FWS, except that very high consumption showed a negative association (Fig. 2).

PRS analyses had adequate power (⩾80%) in the group without lifetime MDD (all PRS), RMDD (MDD, schizophrenia and neuroticism PRS) and SMDD (MDD and schizophrenia PRS only) (online Supplementary Table S6). The PRSs with the largest effect on FWS were MDD, neuroticism and schizophrenia PRS, with similar effect sizes across groups (Fig. 3, online Supplementary Table S5). Interestingly, the only PRS that showed a heterogeneous effect across the three groups was ADHD: this PRS had a stronger effect on FWS in individuals with RMDD (*z* = 2.53, *p* = 0.01) and SMDD (*z* = 2.33, *p* = 0.02) than those without lifetime MDD.

**Fig. 4.**
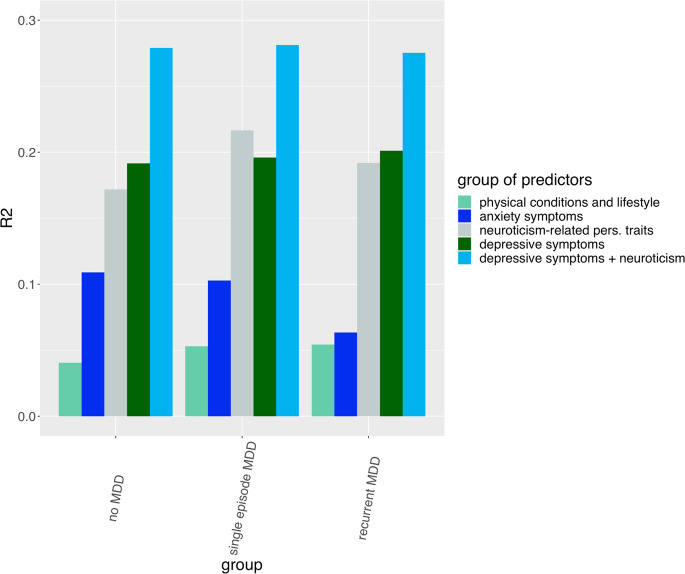
Variance explained (R2) in the observed functioning-wellbeing score (FWS) by combining the predictors in each category using elastic net regression models.

Considering the combined effect of variables in each predictor category, depressive symptoms/characteristics and neuroticism-related personality traits explained the largest proportion of variability in FWS in all groups of individuals (*R*^2^ was 0.19–0.20 for depressive symptoms/characteristics and 0.19–0.22 for neuroticism-related personality traits, Fig. 4, online Supplementary Table S7). The variance in FWS explained by neuroticism-related personality traits was higher in individuals with SMDD compared to the other groups, while the variance explained by depressive symptoms/characteristics was slightly higher in those with RMDD. Adding neuroticism to depressive symptoms/characteristics increased the variance explained in FWS (~0.28 in all groups). The variance in FWS explained by anxiety symptoms/characteristics was higher in those without lifetime MDD and SMDD compared to RMDD (0.11, 0.10 and 0.06, respectively). Lifestyle/physical diseases explained the lowest variance in FWS compared to other variables, with slightly higher values in participants with MDD than in those without MDD (0.05 *v.* 0.04). The variance in FWS explained by PRSs was between 0.0046 and 0.0055 only, though statistically significant (online Supplementary Table S7).

### Mediation analysis

For this analysis, we considered neuroticism as mediator as it is an aggregated measure of the assessed personality traits, it was previously strongly associated with depression and wellbeing (Introduction), and it had a strong association with FWS in UKB (Fig. 2).

Compared to the other variables, neuroticism had higher indirect effects on FWS by acting through the modulation of concentration difficulties and tiredness during the worst depression (percentage mediated 40–49%, and 38–63%, respectively) and the variable ever unenthusiastic for one whole week (percentage mediated 33–44%). The indirect effect of neuroticism on the latter was higher in individuals with lifetime MDD than those without lifetime MDD.

Interestingly, the indirect effect of neuroticism on FWS through the variables ever self-harm, ever contemplated self-harm, impact of depression on normal roles and reactive depression were smaller in those with RMDD compared to participants with SMDD and no MDD (online Supplementary Table S8).

## Discussion

This study provides a comprehensive evaluation of the factors associated with wellbeing in individuals stratified by their history of lifetime MDD. The results are clinically relevant because: (1) the examined variables showed a wide variability in their association with FWS, with effect sizes up to 23 times larger for the top statistically significant variables compared to the others; (2) the cumulative contribution of each category of predictors further elucidated that FWS is mostly influenced by a combination of depressive symptoms and neuroticism-related traits, with indirect effects of the latter on the first; (3) the impact of some predictors depended on the history of MDD, but most predictors showed similar effect sizes across groups.

Contemplated or acted self-harm, chronic depression, loneliness, neuroticism and fed-up feelings had the strongest impact on FWS. Loneliness was previously demonstrated to be among the strongest predictors of poor health in UKB (Mutz, Roscoe, & Lewis, 2021). Worthlessness feelings during the worst depression were also among the top predictors of FWS, consistent with the previous literature (Christensen et al., 2020). Cumulatively, the highest variance in FWS was explained by depressive symptoms/characteristics and neuroticism-related personality traits; the latter showed a larger effect in those with SMDD. Anxious depression and MDD with atypical neurovegetative symptoms were associated with lower FWS, consistent with previous studies, though these did not specifically evaluate wellbeing (Brailean, Curtis, Davis, Dregan, & Hotopf, 2020; Kraus et al., 2019). Depression reactive to a stressful life event showed a positive association with FWS, in accordance with previous evidence of lower depression recurrence in this group (Kessing, 2004). Among the neuroticism-related personality traits, only risk taking was positively associated with wellbeing, in line with a previous study that examined harm avoidance (Spittlehouse et al., 2014).

Anxiety symptoms/characteristics explained more variance in FWS in those without RMDD compared with RMDD, while physical conditions and lifestyle explained the smallest variance in FWS compared to the previously mentioned variables. Interestingly, a previous diagnosis of cancer had a much smaller effect on FWS than chronic obstructive pulmonary disease, diabetes and cardiovascular disorders. Moderate alcohol drinking habits were shown to have a positive association with FWS, in line with the previous literature (Stranges et al., 2014). No or very low alcohol intake was negatively associated with FWS, but might be attributable to individuals not drinking alcohol because of medical conditions that contraindicate it (Wood et al., 2018). Poor health was associated with both current and lifetime alcohol abstinence in a previous study, suggesting that also other factors are involved (Mutz et al., 2021).

To the best of our knowledge, no previous study estimated and compared the contribution of these variables on FWS. However, neuroticism, loneliness, depression and wellbeing were previously shown to be highly related to each other, phenotypically and genetically (Abdellaoui et al., 2019; Baselmans et al., 2019; Jamshidi et al., 2020; Okbay et al., 2016). Interestingly, neuroticism, loneliness, fed-up feelings and chronic depression contributed more evidently to poor FWS in those with SMDD than in the other groups, suggesting a particularly strong influence of neuroticism and related traits in these individuals. Participants with RMDD reported depressive-anxious personality traits more frequently than those with SMDD (online Supplementary Table S4), but as noted, their impact on FWS was larger in those with SMDD. We suggest that individuals with SMDD may have a higher risk of poor wellbeing related to their neuroticism-related traits, rather than the depressive episode and its possible long-term consequences (e.g. residual depressive symptoms). This hypothesis is supported by the finding that neuroticism added higher variance explained in FWS in those with RMDD and no MDD compared to SMDD, suggesting that in SMDD the effects of symptoms reported during the depressive episode and neuroticism are more correlated. Importantly, the three groups were likely to be heterogeneous for many variables, such as environmental exposures, and we could not determine if some of these acted as stratification factors and affected the results.

The high correlation between neuroticism, depressive symptoms and FWS in the previous literature suggests the presence of possible indirect effects of neuroticism on FWS through the modulation of the clinical manifestations of depression (Klein, Kotov, & Bufferd, 2011). It is indeed reasonable to hypothesize that personality traits related to depression-anxiety shape the way individuals develop and perceive symptoms during depression. The largest indirect effect of neuroticism on FWS was found through the modulation of concentration difficulties and tiredness during the worst depressive episode. Previously, these symptoms were found to impact psychosocial functioning in individuals with MDD (Christensen et al., 2020; McIntyre et al., 2013). Other symptoms during the worst depressive episode showing relevant indirect effects of neuroticism were worthlessness feelings and weight gain. However, as previously noted, the cross-sectional design does not identify causal effects, and neuroticism remains partially independent from depressive symptoms, as neuroticism increased the variance in FWS explained by depressive symptoms.

Our genetic analysis showed that the PRS of MDD had the strongest impact on FWS in all groups, followed by the PRSs of neuroticism and schizophrenia, with similar effect sizes independent of MDD history. Previous studies showed strong genetic correlations (~−0.80) between depression, neuroticism and wellbeing (Okbay et al., 2016), while genetic correlations with other psychiatric disorders were lower, in line with our findings (Demontis et al., 2019; Stahl et al., 2019). ADHD PRS had a negative effect on FWS particularly in those with lifetime MDD, suggesting the hypothesis that ADHD predisposition may be more relevant in determining wellbeing in this group. This result is consistent with the previous finding that the ADHD PRS was associated with treatment-resistant depression, a condition that clearly has a negative impact on wellbeing in patients with MDD (Fabbri et al., 2021). We emphasize that other PRSs (including MDD) showed a similar effect size on wellbeing across groups, suggesting that their effect on FWS does not depend on the history of MDD. Schizophrenia and bipolar disorder have a higher genetic correlation with depression than ADHD (Howard et al., 2019), confirming our hypothesis of a lack of a systematic stratification effect depending on MDD diagnosis.

The limitations of the present study should be considered. First, our classification of lifetime MDD was largely based on self-reported information, though previous studies showed a good validity of instruments such as the CIDI-SF (Haro et al., 2006). We note that about half of patients with MDD according to CIDI-SF or Smith et al. definition also had a primary care diagnosis (online Supplementary Table S3), aligning with previous findings that over half of cases of depression are untreated (Briggs, Tobin, Kenny, & Kennelly, 2018). Recurrence of MDD and the variables included in our analyses were also mostly self-reported and could be affected by recall bias or social desirability. The limited overlap between different measures of depression in those with SMDD suggested a larger uncertainty in the diagnosis compared to RMDD (online Supplementary Table S3); it should also be considered that MDD is typically a recurrent disease (Conradi, Bos, Kamphuis, & de Jonge, 2017). We could not exclude the possibility that the answers given to the questions used to determine wellbeing were influenced by depressive symptoms at the time of responding, though some questions were included in the MHQ and individuals with current MDD at the time of the MHQ were excluded. The comparison of the effect of the tested variables on FWS between individuals with remitted and current MDD would have been interesting; however, as noted, it was only partially possible to know the phase of disease each participant was when answering questions included in the FWS. Similarly, answers to the questions evaluating personality may have been affected by depressive-anxiety symptoms at the time the answers were given; however, these questions were formulated to reflect the concept of a stable condition rather than episodic. The available information on personality was limited to neuroticism-related traits; therefore, it was not possible to systematically evaluate other dimensions.

We tried to provide a comprehensive evaluation of the possible determinants of wellbeing, but we acknowledge that other variables could have been considered. However, some of these are likely relevant in a small subset of the sample (e.g. substances other than cannabis, vigorous physical activity), while others are related to the considered predictors (diet is influenced by concomitant physical diseases and social relationships are related with personality, particularly perceived loneliness). When selecting PRSs, we limited the analysis to traits with strong rationale of being involved in the modulation of wellbeing. The power was low for some PRSs in the groups with SMDD and RMDD, although these groups had similar results to the well-powered analysis in those without MDD. The identification of possible mechanisms of the observed differences among individual groups or stratification effects responsible for these differences would be an ambitious objective and was beyond the aims of this study. Finally, UKB is not representative of the general population, with respondents more likely to be older, female, healthier, of a higher socioeconomic background and better educated (Fry et al., 2017) and so our findings should not be extrapolated to general population.

In conclusion, this study provides an estimation of the contribution of depressive and anxiety symptoms and clinical characteristics, neuroticism-related traits, physical conditions, lifestyle and genetic factors to individual wellbeing in participants of UKB, stratified by history of MDD. Our results are relevant for the evaluation of risk factors of poor wellbeing in these groups and for the implementation of appropriate health care or preventive strategies in those at risk.

## Supplementary material

For supplementary material accompanying this paper visit https://doi.org/10.1017/S003329172100502X. click here to view supplementary material
