# Supplementary material for: Depressive symptoms and neuroticism-related traits are the main factors associated with wellbeing independent of the history of lifetime depression in the UK Biobank
Source: Psychol Med. 2021 Dec 9;53(7):3000–8. doi: 10.1017/S003329172100502X (PMC10235644; doi:10.1017/S003329172100502X)

Depressive symptoms and neuroticism-related traits are the main factors associated with wellbeing independent of the history of lifetime depression in the UK Biobank

Supplementary Materials

Table of contents

Supplementary Methods.. p. 1
Supplementary Table 1.. p. 4
Supplementary Table 2.. p. 9
Supplementary Table 3.. p. 10
Supplementary Table 4.. p. 11
Supplementary Table 5.. p. 16
Supplementary Table 6.. p. 21
Supplementary Table 7.. p. 22
Supplementary Table 8.. p. 23
Supplementary Figure 1.. p. 25
Supplementary Figure 2.. p. 27
Supplementary Figure 3.. p. 28

Supplementary Methods

UK Biobank sample

Individuals registered with the UK National Health Service (NHS) and living within a 25-mile (~40 km) radius of one of 22 assessment centres were invited to participate. Participants provided information on their sociodemographic characteristics, lifestyle and medical history. They underwent physical examination including height, weight and blood pressure measurement. They also provided blood, urine and saliva samples, which were stored in such a way as to allow many different types of assay to be performed (for example, genetic, proteomic and metabonomic analyses). Repeat assessments of baseline information have been conducted in subsets of the cohort every few years, to enable calibration of measurements, adjustment for regression dilution, and estimation of longitudinal change.

All participants provided consent for follow-up through linkage to their health-related records. Linked hospital inpatient records are available for most participants and primary care records are currently available for about half of participants. A subset of 157,366 out of 339,092 invited participants (46%) completed an online follow-up mental health questionnaire (MHQ) between 2016 and 2017, covering 31% of all participants. Further information on UK Biobank was reported elsewhere (Bycroft et al., 2018).

Genotyping, quality control and imputation

Genome-wide genotyping on all UK Biobank participants was performed using two highly overlapping arrays covering ~800,000 markers. Autosomal genotype data underwent centralized quality control to adjust for possible array effects, batch effects, plate effects, and departures from Hardy-Weinberg equilibrium (HWE) (Bycroft et al., 2018). SNPs were further excluded based on missingness (> 0.02) and on Hardy Weinberg equilibrium (p<10-8). Individuals were removed for high levels of missingness (> 0.05) or abnormal heterozygosity (as defined during centralised quality control), relatedness of up to third-degree kinship (KING r < 0.044 (Manichaikul et al., 2010)) or phenotypic and genotypic gender discordance. Population structure within the UK Biobank cohort

was assessed using principal component analysis, with European ancestry defined by 4-means clustering on the first two genetic principal components (Warren et al., 2017).
A two-stage imputation was performed using the Haplotype Reference Consortium (HRC) and UK10K reference panels (Bycroft et al., 2018) (McCarthy et al., 2016) (UK10K Consortium et al., 2015). Poor imputed variants were excluded (INFO ≤ 0.4) (McCarthy et al., 2016).

Creation of the functional-wellbeing score: comparison with a factor analysis

As only four variables with medium-strong correlations (Figure 1) were used to create the functional-wellbeing score (FWS), we opted for a simple averaging approach rather than more complex methods. However, for completeness and as a check, we compared the FWS obtained by the averaging approach described in paragraph "Measurement of functioning and wellbeing" of the main text with that obtained by a factor analysis.
We used the full information maximum likelihood (FIML) method to perform an exploratory factor analysis, as implemented in the R library 'umx' (Bates, Maes, & Neale, 2019), to account for the presence of missing data (individuals having ≥ 2 non-missing input variables were considered, as done for the averaging method). Using the Kaiser rule (Kaiser, 1960), parallel analysis (Horn, 1965) and identification of an elbow in the scree plot, as implemented in the R library 'nFactors', we determined that one factor was sufficient to represent the four variables (Supplementary Figure 2). The results showed that loadings were 0.86 for happiness and 0.68 for social/work satisfaction, while 0.60 for life meaningfulness and 0.47 for health satisfaction; the identified factor showed a Pearson's correlation coefficient of 0.9197 (95% confidence interval: 0.9191 - 0.9203) with the FWS calculated by averaging the four variables of interest.
Therefore, we suggest that the use of a factor analysis to estimate the FWS would not represent a relevant improvement in the methodology of the study and would not lead to relevant differences in the results, as only four variables were considered to build the FWS, they had medium-strong correlations among each other, only one factor was sufficient and it was highly correlated with the FWS obtained by simply averaging the four variables.

References

Bates, T. C., Maes, H., & Neale, M. C. (2019). umx: Twin and Path-Based Structural Equation Modeling in R. *Twin Research and Human Genetics*, *22*(1), 27–41. https://doi.org/10.1017/thg.2019.2

Bycroft, C., Freeman, C., Petkova, D., Band, G., Elliott, L. T., Sharp, K., … Marchini, J. (2018). The UK Biobank resource with deep phenotyping and genomic data. *Nature*, *562*(7726), 203–209. https://doi.org/10.1038/s41586-018-0579-z

Fabbri, C., Hagenaars, S. P., John, C., Williams, A. T., Shrine, N., Moles, L., … Lewis, C. M. (2021). Genetic and clinical characteristics of treatment-resistant depression using primary care

records in two UK cohorts. *Molecular Psychiatry*, *26*(7), 3363–3373. https://doi.org/10.1038/s41380-021-01062-9

Horn, J. L. (1965). A rationale and test for the number of factors in factor analysis. *Psychometrika*, *30*(2), 179–185. https://doi.org/10.1007/BF02289447

Kaiser, H. F. (1960). The Application of Electronic Computers to Factor Analysis. *Educational and Psychological Measurement*, *20*(1), 141–151. https://doi.org/10.1177/001316446002000116

Manichaikul, A., Mychaleckyj, J. C., Rich, S. S., Daly, K., Sale, M., & Chen, W.-M. (2010). Robust relationship inference in genome-wide association studies. *Bioinformatics (Oxford, England)*, *26*(22), 2867–2873. https://doi.org/10.1093/bioinformatics/btq559

McCarthy, S., Das, S., Kretzschmar, W., Delaneau, O., Wood, A. R., Teumer, A., … Haplotype Reference Consortium. (2016). A reference panel of 64,976 haplotypes for genotype imputation. *Nature Genetics*, *48*(10), 1279–1283. https://doi.org/10.1038/ng.3643

UK10K Consortium, Walter, K., Min, J. L., Huang, J., Crooks, L., Memari, Y., … Soranzo, N. (2015). The UK10K project identifies rare variants in health and disease. *Nature*, *526*(7571), 82–90. https://doi.org/10.1038/nature14962

Warren, H. R., Evangelou, E., Cabrera, C. P., Gao, H., Ren, M., Mifsud, B., … UK Biobank CardioMetabolic Consortium BP working group. (2017). Genome-wide association analysis identifies novel blood pressure loci and offers biological insights into cardiovascular risk. *Nature Genetics*, *49*(3), 403–415. https://doi.org/10.1038/ng.3768

Supplementary Table 1: variables tested for association with functioning/wellbeing. Categorical variables were recoded to dummy variables or binary variables as described.

Category	Description	UKB Data field or definition	Variable coding/information
Depressive symptoms and characteristics of depression	Age at depression onset	20433 or age at first record of depression diagnosis in primary care if 20433 was missing	Continuous
	Number of depression episodes	4620	Continuous
	Duration of worst depression	20438	6 levels, from less than a month (1) to over 2 years (6); recoded to dummy variables with reference level 1
	Fraction of the day affected during worst episode of depression	20436	4 levels, from less than half (1) of the day to all day long (4); recoded to binary variable (less than half of the day or about half of the day=0; most of the day or all day long=1)
	Frequency of depressed days during worst episode of depression	20439	3 levels (1=less often, 2=almost every day, 3=every day); recoded to binary variable (1=almost every day/every day; 0=less often)
	Impact on normal roles during worst episode of depression	20440	4 levels, from not at all (0) to a lot (3); recoded to binary variable (not at all and a little=0; somewhat and a lot=1)
	Ever unenthusiastic /disinterested for a whole week	4631	Binary
	Ever had prolonged loss of interest in normal activities	20441	Binary
	Ever contemplated self-harm	20485	3 levels (0=no, 1=once, 2=more than once), recoded as binary (0=no, 1=yes)

	Ever self-harmed	20480	Binary
	Ever attempted suicide	20483	Binary
	Thoughts of death during worst depression	20437	Binary
	Feelings of worthlessness during worst period of depression	20450	Binary
	Feelings of tiredness during worst episode of depression	20449	Binary
	Difficulty concentrating during worst depression	20435	Binary
	Did your sleep change? (referred to depression)	20532	Binary
	Was that: [re sleep change] sleeping too much?	20534	Binary
	Was that: [re sleep change] waking too early?	20535	Binary
	Weight change during worst episode of depression	20536	4 levels: 0=no, 1=gained weight, 2=lost weight, 3=both 1 and 2; recoded as dummy variable where the reference was 0
	Depression possibly related to stressful or traumatic event	20447	Binary
	Anxious depression	Wake too early + at least two of the following: tense/ 'highly strung', worrier/anxious feelings, nervous feelings	Binary
	Depression with atypical symptoms	Weight/appetite gain + sleeping too much	Binary
	Depression with typical symptoms	Weight/appetite loss + waking too early	Binary

	Treatment-resistant depression	Defined according to (Fabbri et al., 2021)	Binary
Anxiety symptoms and characteristics of anxiety	Ever felt worried, tense, or anxious for most of a month or longer	20421	Binary
	Worried most days during period of worst anxiety	20538	Binary
	Impact on normal roles during worst episode of anxiety	20418	4 levels, from not at all (0) to a lot (3); recoded to binary variable (not at all and a little=0; somewhat and a lot=1)
	Difficulty concentrating during worst period of anxiety	20419	Binary
	Difficulty stopping worrying during worst period of anxiety	20541	Binary
	Easily tired during worst period of anxiety	20429	Binary
	Frequent trouble falling or staying asleep during worst period of anxiety	20427	Binary
	Keyed up or on edge during worst period of anxiety	20423	Binary
	More irritable than usual during worst period of anxiety	20422	Binary
	Multiple worries during worst period of anxiety	20540	Binary
	Restless during period of worst anxiety	20426	Binary
	Tense, sore, or aching muscles during worst period of anxiety	20417	Binary

	Stronger worrying (than other people) during period of worst anxiety	20542	Binary
	Ever worried more than most people would in similar situation	20425	Binary
Neuroticism-related personality traits	Worry too long after embarrassment	2000	Binary
	Sensitivity/hurt feelings	1950	Binary
	Worrier/anxious feelings	1980	Binary
	Nervous feelings	1970	Binary
	Guilty feelings	2030	Binary
	Fed-up feelings	1960	Binary
	Tense/'highly strung'	1990	Binary
	Suffer from 'nerves'	2010	Binary
	Miserableness	1930	Binary
	Loneliness	2020	Binary
	Mood swings	1920	Binary
	Irritability	1940	Binary
	Risk taking	2040	Binary
	Neuroticism score	20127	Continuous
Physical conditions, cannabis and alcohol use	Long-standing illness, disability or infirmity	2188	Binary
	Cancer diagnosed by doctor	2453	Binary
	Diabetes diagnosed by doctor	2443	Binary
	Emphysema/chronic bronchitis diagnosed by doctor	Extracted from 6152	Binary
	Vascular/heart problems diagnosed by doctor (heart attack, angina and stroke; high	Extracted from 6150	Binary

	blood pressure was not considered)		
	BMI	21001	Continuous
	Number of days/week of moderate physical activity	884	From 0 to 7
	Ever taken cannabis	20453	5 levels, from no (0) to more than 100 times (4), recoded to binary variable (< 10 times vs > 10 times)
	Amount of alcohol drunk on a typical drinking day	20403	5 levels, from 1 or 2 (1) to 10 or more (5), recoded to dummy variables with reference level 1
	Alcohol intake frequency	1558	6 levels, from never (6) to daily or almost daily (1), recoded to dummy variables with reference level 3 (1-2 times per week)
	Frequency of consuming six or more units of alcohol	20416	5 levels, from never (1) to daily or almost daily (5), recoded to dummy variables with reference level 1
Polygenic risk scores (PRS)	PRS of schizophrenia	PubMed ID 29483656	40,675 cases and 64,643 controls
	PRS of MDD	PubMed ID 29700475 (excluding UKB and 23andMe)	45,591 cases and 97,674 controls
	PRS of bipolar disorder	PubMed ID 31043756	20,352 cases and 31,358 controls
	PRS of attention-deficit hyperactivity disorder	PubMed ID 30478444	20,183 cases and 35,191 controls
	PRS of alcohol dependence	PubMed ID 30482948	14,904 cases and 37,944 controls
	PRS of anxiety disorders	PubMed ID 26754954	7,016 cases and 14,745 controls
	PRS of neuroticism	PubMed ID 25993607	Continous measure in 63,030 adults

Supplementary Table 2: clinical-demographic characteristics of individuals with lifetime recurrent MDD (RMDD), single episode MDD (SMDD) and no MDD. A greater Townsend deprivation index implies a greater degree of deprivation. FWS=functioning-wellbeing score.

Variable	RMDD (n=47,966)	SMDD (n=21,117)	No MDD (n=207,423)
Age at assessment	55.09±7.81	56.10±7.89	56.74±8.00
Sex F/M	32,584(68%)/15,382	13,630(65%)/7,487	105,689(51%)/101,734
Townsend deprivation index	-1.15±3.02	-1.32±2.95	-1.51±2.90
Happiness	4.26±0.72	4.43±0.72	4.58±0.68
Life meaningfulness	3.96±1.17	4.36±1.12	4.75±0.94
Health satisfaction	4.05±0.93	4.16±0.94	4.38±0.82
Social/work satisfaction	4.51±0.70	4.65±0.69	4.73±0.63
FWS	4.19±0.66	4.37±0.66	4.57±0.58

Supplementary Table 3: distribution of different depression measures in those with recurrent MDD and single episode MDD (**A**) and overlap between the different depression measures (**B**).

A

**Measure**	**Recurrent MDD (n=47,966)**	**Single episode MDD (n=21,117)**
Smith depression	23,727	6,209
CIDI-SF depression	23,516	9,061
ICD depression	4,960	2,666
Primary care depression	13,422	5,780

B

Type of MDD	Measure to define depression	Other measure of depression	N overlap/N total (% overlap)
Recurrent MDD	Smith depression	CIDI-SF	4880/7272 (67.11)
		ICD	2465/19577 (12.59)
		Primary care	5479/10825 (50.61)
	CIDI-SF	Smith depression	4880/5870 (83.13)
		ICD	1544/18843 (8.19)
		Primary care	4234/9962 (42.50)
	ICD	CIDI-SF	1544/1890 (81.69)
		Smith depression	2465/2659 (92.70)
		Primary care	2028/2520 (80.48)
	Primary care	ICD	2028/11362 (17.85)
		CIDI-SF	4234/5839 (72.51)
		Smith depression	5479/6464 (84.76)
Single episode MDD	Smith depression	CIDI-SF	581/1521 (38.20)
		ICD	197/5089 (3.87)
		Primary care	645/2548 (25.31)
	CIDI-SF	Smith depression	581/1301 (44.66)
		ICD	250/7164 (3.49)
		Primary care	639/3541 (18.05)
	ICD	CIDI-SF	250/509 (49.12)
		Smith depression	197/912 (21.60)
		Primary care	430/888 (48.42)
	Primary care	ICD	430/4882 (8.81)
		CIDI-SF	639/1741 (36.70)
		Smith depression	645/2462 (26.20)

Supplementary Table 4: distribution of the variables of interest in individuals with recurrent MDD, single episode MDD and no history of MDD. MDD=major depressive disorder. **A**: distribution of binary variables; **B**: distribution of categorical variables; **C**: description of continuous variables. RMDD=recurrent major depressive disorder. SMDD=single episode major depressive disorder. Pr.=proportion. Dep.=depression. Anx.=anxiety.

A

Variable	group	yes	no	pr. yes	pr. missing	group	yes	no	pr. yes	pr. missing	group	yes	no	pr. yes	pr. missing
ever self harm	RMDD	2871	26667	0.0972	0.3842	SMDD	674	11288	0.0563	0.4335	no MDD	1955	106965	0.0179	0.4749
ever attempted suicide	RMDD	1538	1240	0.5536	0.9421	SMDD	391	260	0.6006	0.9692	no MDD	771	1132	0.4051	0.9908
ever contemplated self harm	RMDD	9646	19876	0.3267	0.3845	SMDD	2435	9493	0.2041	0.4351	no MDD	7997	100817	0.0735	0.4754
most of the day affected during worst dep.	RMDD	26220	2208	0.9223	0.4073	SMDD	9915	613	0.9418	0.5014	no MDD	19313	19230	0.5011	0.8142
depressed every day or almost during worst dep.	RMDD	27877	782	0.9727	0.4025	SMDD	10384	254	0.9761	0.4962	no MDD	33146	7774	0.8100	0.8027
impact on normal roles during worst dep.	RMDD	26234	2597	0.9099	0.3989	SMDD	9928	815	0.9241	0.4913	no MDD	16773	26430	0.3882	0.7917
worthlessness during worst dep.	RMDD	18700	8357	0.6911	0.4359	SMDD	5726	4292	0.5716	0.5256	no MDD	13160	27057	0.3272	0.8061
gained weight vs no change during worst dep.	RMDD	5371	7184	0.4278	0.7383	SMDD	1436	2784	0.3403	0.8002	no MDD	4776	19314	0.1983	0.8839
lost weight vs no change during worst dep.	RMDD	10854	7184	0.6017	0.6239	SMDD	4317	2784	0.6079	0.6637	no MDD	10028	19314	0.3418	0.8585
both gained and lost weight vs no change during worst dep.	RMDD	1908	7184	0.2099	0.8104	SMDD	529	2784	0.1597	0.8431	no MDD	1766	19314	0.0838	0.8984
sleep change during worst dep.	RMDD	23611	2604	0.9007	0.4535	SMDD	8552	997	0.8956	0.5478	no MDD	23692	11325	0.6766	0.8312
waking too early during worst dep.	RMDD	18193	5418	0.7705	0.5078	SMDD	6344	2208	0.7418	0.5950	no MDD	17719	5973	0.7479	0.8858
sleep too much during worst dep.	RMDD	6040	17571	0.2558	0.5078	SMDD	1786	6766	0.2088	0.5950	no MDD	3000	20692	0.1266	0.8858
tiredness during worst dep.	RMDD	25442	1862	0.9318	0.4308	SMDD	9178	862	0.9141	0.5246	no MDD	25447	11323	0.6921	0.8227
concentration difficulties during worst dep.	RMDD	24887	2079	0.9229	0.4378	SMDD	9095	808	0.9184	0.5310	no MDD	22365	13489	0.6238	0.8271
ever prolonged loss of interest in normal activities	RMDD	25258	4385	0.8521	0.3820	SMDD	9048	2950	0.7541	0.4318	no MDD	22122	86626	0.2034	0.4757
ever unenthusiastic for a whole week	RMDD	23918	6713	0.7808	0.3614	SMDD	6541	5636	0.5372	0.4234	no MDD	26482	89725	0.2279	0.4398
lifetime dep. with atypical symptoms	RMDD	1372	28308	0.0462	0.3812	SMDD	330	11690	0.0275	0.4308	-	-	-	-	-

lifetime dep. with typical symptoms	RMDD	8048	21632	0.2712	0.3812	SMDD	3202	8818	0.2664	0.4308	-	-	-	-	-
lifetime anxious dep.	RMDD	6571	17040	0.2783	0.5078	SMDD	1872	6680	0.2189	0.5950	-	-	-	-	-
dep. possibly related to stressful or traumatic event	RMDD	20873	7904	0.7253	0.4001	SMDD	8151	2564	0.7607	0.4926	no MDD	31933	11264	0.7392	0.7917
treatment-resistant depression (primary care data)	RMDD	1059	7332	0.1262	0.8251	SMDD	63	1072	0.0555	0.9463	-	-	-	-	-
death thoughts during worst dep.	RMDD	16757	10455	0.6158	0.4327	SMDD	5508	4614	0.5442	0.5207	no MDD	17205	23529	0.4224	0.8036
chronic depression	RMDD	3564	25077	0.1244	0.4029	SMDD	712	9883	0.0681	0.4983	no MDD	2591	39807	0.0611	0.7956
restless during worst anx.	RMDD	11280	5778	0.6613	0.6444	SMDD	2845	1649	0.6331	0.7872	no MDD	9677	9603	0.5019	0.9070
concentration difficulties during worst anx.	RMDD	15115	2480	0.8591	0.6332	SMDD	3809	860	0.8158	0.7789	no MDD	12091	7677	0.6116	0.9047
difficulty stop worring during worst anx.	RMDD	17424	529	0.9705	0.6257	SMDD	4575	207	0.9567	0.7735	no MDD	18090	2022	0.8995	0.9030
easily tired during worst anx.	RMDD	13982	3338	0.8073	0.6389	SMDD	3476	1087	0.7618	0.7839	no MDD	11545	7704	0.5998	0.9072
ever worried/tense/anxious for a month or longer	RMDD	15876	10724	0.5968	0.4454	SMDD	4153	6611	0.3858	0.4903	no MDD	15123	88798	0.1455	0.4990
ever worried more than most people in similar situation	RMDD	12856	10581	0.5485	0.5114	SMDD	3338	6220	0.3492	0.5474	no MDD	14461	79282	0.1543	0.5481
trouble sleeping during worst anx.	RMDD	15499	2394	0.8662	0.6270	SMDD	4041	730	0.8470	0.7741	no MDD	15278	5017	0.7528	0.9022
impact on normal roles during worst anx.	RMDD	14732	3543	0.8061	0.6190	SMDD	3662	1224	0.7495	0.7686	no MDD	9308	11615	0.4449	0.8991
keyed up/on edge during worst anx.	RMDD	14751	2774	0.8417	0.6346	SMDD	3819	846	0.8186	0.7791	no MDD	14072	5701	0.7117	0.9047
irratable during worst anx.	RMDD	12958	3874	0.7698	0.6491	SMDD	3269	1163	0.7376	0.7901	no MDD	12214	6814	0.6419	0.9083
multiple worries during worst anx.	RMDD	14633	3039	0.8280	0.6316	SMDD	3522	1165	0.7514	0.7780	no MDD	13174	6724	0.6621	0.9041
stronger worrying than other people during worst anx.	RMDD	11284	1698	0.8692	0.7293	SMDD	2717	554	0.8306	0.8451	no MDD	10405	3477	0.7495	0.9331
tense/sore/aching muscles during worst anx.	RMDD	7654	8875	0.4631	0.6554	SMDD	1693	2683	0.3869	0.7928	no MDD	5663	13659	0.2931	0.9068
worried most days during worst anx.	RMDD	16579	1252	0.9298	0.6283	SMDD	4270	441	0.9064	0.7769	no MDD	16279	3655	0.8166	0.9039
ever taken cannabis less than 10 times vs more times	RMDD	2789	26840	0.0941	0.3823	SMDD	886	11125	0.0738	0.4312	no MDD	6616	102352	0.0607	0.4747
fed up feelings	RMDD	26776	20398	0.5676	0.0165	SMDD	9637	11040	0.4661	0.0208	no MDD	66041	136834	0.3255	0.0219

loneliness feelings	RMDD	14354	32797	0.3044	0.0170	SMDD	4673	16073	0.2252	0.0176	no MDD	26864	177420	0.1315	0.0151
miserableness	RMDD	30622	16769	0.6462	0.0120	SMDD	10629	10165	0.5112	0.0153	no MDD	70821	133040	0.3474	0.0172
irritability	RMDD	17459	28603	0.3790	0.0397	SMDD	6404	13743	0.3179	0.0459	no MDD	48791	149748	0.2458	0.0428
sensitivity hurt feelings	RMDD	32608	14333	0.6947	0.0214	SMDD	12661	7863	0.6169	0.0281	no MDD	100027	101061	0.4974	0.0305
guilty feelings	RMDD	20452	26479	0.4358	0.0216	SMDD	6986	13560	0.3400	0.0270	no MDD	47604	154309	0.2358	0.0266
suffer from 'nerves'	RMDD	15242	30695	0.3318	0.0423	SMDD	5007	15187	0.2479	0.0437	no MDD	31665	168971	0.1578	0.0327
tense/highly strung	RMDD	13183	33082	0.2849	0.0355	SMDD	4233	16080	0.2084	0.0381	no MDD	25697	174996	0.1280	0.0324
worry too long after embarrassment	RMDD	28627	17842	0.6160	0.0312	SMDD	10727	9565	0.5286	0.0391	no MDD	87522	111111	0.4406	0.0424
worrier/anxious feelings	RMDD	33540	13477	0.7134	0.0198	SMDD	13102	7517	0.6354	0.0236	no MDD	101162	100502	0.5016	0.0278
nervous feelings	RMDD	15669	31057	0.3353	0.0259	SMDD	5589	14896	0.2728	0.0299	no MDD	38085	164327	0.1882	0.0242
mood swings	RMDD	30336	16959	0.6414	0.0140	SMDD	10718	9917	0.5194	0.0228	no MDD	73857	128302	0.3653	0.0254
risk taking	RMDD	13386	32971	0.2888	0.0335	SMDD	5231	15044	0.2580	0.0399	no MDD	52817	146833	0.2645	0.0375
COPD	RMDD	824	47089	0.0172	0.0011	SMDD	379	20697	0.0180	0.0019	no MDD	2139	204904	0.0103	0.0018
Vascular/heart problems diagnosed by doctor	RMDD	2380	45499	0.0497	0.0018	SMDD	1207	19868	0.0573	0.0020	no MDD	9652	197286	0.0466	0.0023
Diabetes diagnosed by doctor	RMDD	2292	45554	0.0479	0.0025	SMDD	1094	19957	0.0520	0.0031	no MDD	9582	197252	0.0463	0.0028
Longstanding illness, disability or infirmity	RMDD	18556	28272	0.3963	0.0237	SMDD	7479	13115	0.3632	0.0248	no MDD	55829	147090	0.2751	0.0217
Cancer diagnosed by doctor	RMDD	3900	43907	0.0816	0.0033	SMDD	1740	19289	0.0827	0.0042	no MDD	15031	191749	0.0727	0.0031

B

variable	group	distribution	pr. missing	group	distribution	pr. missing	group	distribution	pr. missing
duration of worst dep.	RMDD	< 1 month = 1817 (0.0634)<br>1-3 months = 7446 (0.2600)<br>3-6 months = 5849 (0.2042)<br>6-12 months = 5640 (0.1969)<br>1-2 years = 4325 (0.1510)<br>over 2 years = 3564 (0.1244)	0.4029	SMDD	< 1 month = 1232 (0.1163)<br>1-3 months = 3837 (0.3622)<br>3-6 months = 2219 (0.2094)<br>6-12 months = 1709 (0.1613)<br>1-2 years = 886 (0.0836)<br>over 2 years = 712 (0.0672)	0.4983	no MDD	< 1 month = 9931 (0.2342)<br>1-3 months = 14622 (0.3449)<br>3-6 months = 6731 (0.1588)<br>6-12 months = 5143 (0.1213)<br>1-2 years = 3380 (0.0797)<br>over 2 years = 2591 (0.0611)	0.7956
units of alcohol on a typical drinking day	RMDD	1 or 2 = 14044 (0.5264)<br>3 or 4 = 7088 (0.2657)<br>5 or 6 = 3073 (0.1152)<br>7-9 = 1743 (0.0653)<br>10 or more = 732 (0.0274)	0.4438	SMDD	1 or 2 = 5836 (0.5420)<br>3 or 4 = 2825 (0.2624)<br>5 or 6 = 1198 (0.1113)<br>7-9 = 619 (0.0575)<br>10 or more = 289 (0.0268)	0.4901	no MDD	1 or 2 = 51495 (0.5130)<br>3 or 4 = 27837 (0.2773)<br>5 or 6 = 12105 (0.1206)<br>7-9 = 6327 (0.0630)<br>10 or more = 2608 (0.0260)	0.5161
alcohol intake frequency	RMDD	daily or almost = 3880 (0.0809)<br>3-4 times/week = 6088 (0.1300)<br>1-2 times/week = 6239 (0.1301)<br>1-3 times/month = 11726 (0.2446)<br>special occasions = 10577 (0.2206)<br>never = 9430 (0.1967)	0.0005	SMDD	daily or almost = 1713 (0.0812)<br>3-4 times/week = 2600 (0.1233)<br>1-2 times/week = 2599 (0.1232)<br>1-3 times/month = 5371 (0.2547)<br>special occasions = 4692 (0.2225)<br>never = 4114 (0.1951)	0.001	no MDD	daily or almost = 14602 (0.0705)<br>3-4 times/week = 21237 (0.1025)<br>1-2 times/week = 21944 (0.1059)<br>1-3 times/month = 52922 (0.2554)<br>special occasions = 51329 (0.2477)<br>never = 45202 (0.2181)	0.0009
frequency of consuming six or more units of alcohol	RMDD	never = 13837 (0.5178)<br>less than montly = 6541 (0.2448)<br>monthly = 2186 (0.0818)<br>weekly = 3177 (0.1189)<br>daily or almost = 982 (0.0367)	0.4429	SMDD	never = 5758 (0.5331)<br>less than montly = 2597 (0.2404)<br>monthly = 868 (0.0804)<br>weekly = 1246 (0.1154)<br>daily or almost = 332 (0.0307)	0.4885	no MDD	never = 50449 (0.5015)<br>less than montly = 24625 (0.2448)<br>monthly = 9047 (0.0899)<br>weekly = 13294 (0.1322)<br>daily or almost = 3165 (0.0315)	0.5151

C

variable	group	mean	SD	pr. missing	group	mean	SD	pr. missing	group	mean	SD	pr. missing
age at depression onset	RMDD	36.24	13.84	0.3322	SMDD	44.22	12.91	0.5243	no MDD	/	/	/
number of episodes of dep.	RMDD	4.66	6.17	0.1727	SMDD	/	/	/	no MDD	/	/	/
Neuroticism score	RMDD	5.85	3.29	0.1759	SMDD	4.70	3.28	0.1978	no MDD	3.41	2.96	0.1853
Number of days/week of moderate physical activity	RMDD	3.57	2.32	0.0329	SMDD	3.57	2.35	0.0527	no MDD	3.63	2.30	0.0434

Supplementary Table 5: associations between the functioning-wellbeing score (FWS) and the variables of interest. **A**: depressive symptoms/characteristics. **B**: anxiety symptoms/characteristics. **C**: neuroticism-related personality traits. **D**: lifestyle and physical diseases; **E**: polygenic risk scores (PRS). Significant p values are reported in bold (Bonferroni corrected alpha value=2.7e-4 for clinical and socio-demographic variables; Bonferroni corrected alpha value=2.4e-3 for PRS). Dep.=depression. Anx.=anxiety.

A

variable	group	beta	SE	p	group	beta	SE	p	group	beta	SE	p
age at dep. onset	RMDD	0.08038	0.00792	**3.82E-24**	SMDD	-0.02124	0.01468	0.14799925	no MDD	/	/	/
n dep. episodes	RMDD	-0.19018	0.00617	**7.36E-206**	SMDD	/	/	/	no MDD	/	/	/
ever self-harm	RMDD	-0.30826	0.01280	**7.87E-127**	SMDD	-0.37743	0.02681	**1.17E-44**	no MDD	-0.43009	0.01340	**4.92E-225**
ever attempted suicide	RMDD	-0.08562	0.02540	0.000757839	SMDD	-0.05284	0.05946	0.3744821	no MDD	-0.17497	0.03216	**5.97E-08**
ever contemplated self-harm	RMDD	-0.38993	0.00788	**<5e-324**	SMDD	-0.47387	0.01489	**1.78E-213**	no MDD	-0.53976	0.00667	**<5e-324**
worthlessness during worst dep.	RMDD	-0.34316	0.00852	**<5e-324**	SMDD	-0.34734	0.01353	**8.08E-141**	no MDD	-0.40312	0.00647	**<5e-324**
gained weight vs no change during worst dep.	RMDD	-0.18569	0.01188	**1.60E-54**	SMDD	-0.20101	0.02290	**2.41E-18**	no MDD	-0.26158	0.01021	**7.85E-143**
lost weight vs no change during worst dep.	RMDD	0.02881	0.00977	0.003202683	SMDD	0.06610	0.01606	**3.92E-05**	no MDD	0.03166	0.00757	**2.90E-05**
both gained and lost weight vs no change during worst dep.	RMDD	-0.17200	0.01665	**7.20E-25**	SMDD	-0.19222	0.03280	**5.07E-09**	no MDD	-0.20695	0.01542	**6.47E-41**
sleep change during worst dep.	RMDD	0.00586	0.01360	0.666444206	SMDD	-0.01748	0.02290	0.44531126	no MDD	-0.05896	0.00725	**4.24E-16**
waking too early during worst dep.	RMDD	0.00697	0.01018	0.493480518	SMDD	-0.03286	0.01683	0.05090423	no MDD	-0.05754	0.00941	**9.76E-10**
sleep too much during worst dep.	RMDD	-0.14485	0.00983	**5.93E-49**	SMDD	-0.16058	0.01814	**1.04E-18**	no MDD	-0.19663	0.01230	**3.19E-57**
tiredness during worst dep.	RMDD	-0.09800	0.01575	**4.92E-10**	SMDD	-0.10435	0.02428	**1.75E-05**	no MDD	-0.17114	0.00709	**1.14E-127**
concentration difficulties during worst dep.	RMDD	-0.08854	0.01503	**3.92E-09**	SMDD	-0.10918	0.02510	**1.37E-05**	no MDD	-0.15318	0.00688	**6.07E-109**
ever prolonged loss of interest in normal activities	RMDD	-0.26201	0.01067	**6.55E-132**	SMDD	-0.22650	0.01432	**8.81E-56**	no MDD	-0.34540	0.00432	**<5e-324**
ever unenthusiastic for a whole week	RMDD	-0.22297	0.00844	**3.60E-152**	SMDD	-0.19277	0.01090	**4.35E-69**	no MDD	-0.26665	0.00372	**<5e-324**
lifetime dep. with atypical symptoms	RMDD	-0.21540	0.01813	**1.82E-32**	SMDD	-0.33541	0.03796	**1.12E-18**	no MDD	/	/	/
lifetime dep. with typical symptoms	RMDD	0.08104	0.00855	**2.66E-21**	SMDD	0.08479	0.01400	**1.43E-09**	no MDD	/	/	/
lifetime anxious dep.	RMDD	-0.20138	0.00945	**1.02E-99**	SMDD	-0.27883	0.01755	**5.03E-56**	no MDD	/	/	/

dep. possibly related to stressful or traumatic event	RMDD	0.10691	0.00864	**4.76E-35**	SMDD	0.13025	0.01538	**2.77E-17**	no MDD	0.18896	0.00685	**3.76E-166**
treatment-resistant depression	RMDD	-0.28215	0.02212	**6.59E-37**	SMDD	-0.36900	0.08308	**9.81E-06**	no MDD	/	/	/
death thoughts during worst dep.	RMDD	-0.28554	0.00801	**9.20E-272**	SMDD	-0.28001	0.01331	**3.45E-96**	no MDD	-0.19151	0.00622	**1.46E-205**
most of the day affected during worst dep.	RMDD	-0.02341	0.01456	0.107811186	SMDD	-0.02132	0.02842	0.4532441	no MDD	-0.02771	0.00644	**1.69E-05**
depressed every day or almost during worst dep.	RMDD	-0.01570	0.02380	0.509525509	SMDD	0.01224	0.04335	0.7776419	no MDD	0.01250	0.00793	0.11482319
impact on normal roles during worst dep.	RMDD	-0.10308	0.01350	**2.30E-14**	SMDD	-0.08868	0.02490	0.0003708	no MDD	-0.09112	0.00619	**6.28E-49**
chronic dep.	RMDD	-0.25525	0.01164	**8.84E-106**	SMDD	-0.49355	0.02598	**3.46E-79**	no MDD	-0.32473	0.01264	**1.85E-144**

B

**variable**	**group**	**beta**	**SE**	**p**	**group**	**beta**	**SE**	**p**	**group**	**beta**	**SE**	**p**
restless during worst anx.	RMDD	-0.0150	0.0107	0.162824306	SMDD	-0.0287	0.0223	0.1984867	no MDD	-0.0920	0.0094	**1.01E-22**
concentration difficulties during worst anx.	RMDD	-0.1386	0.0144	**6.68E-22**	SMDD	-0.1445	0.0272	**1.17E-07**	no MDD	-0.2225	0.0094	**1.45E-121**
difficulty stop worring during worst anx.	RMDD	-0.2116	0.0292	**4.13E-13**	SMDD	-0.2811	0.0512	**4.20E-08**	no MDD	-0.2631	0.0152	**1.08E-66**
easily tired during worst anx.	RMDD	-0.1610	0.0127	**1.50E-36**	SMDD	-0.2266	0.0250	**1.58E-19**	no MDD	-0.2389	0.0095	**4.24E-138**
ever worried/tense/anxious for a month or longer	RMDD	-0.2083	0.0080	**7.76E-146**	SMDD	-0.2015	0.0131	**8.07E-53**	no MDD	-0.2714	0.0050	**<5e-324**
ever worried more than most people in similar situation	RMDD	-0.3080	0.0084	**4.37E-287**	SMDD	-0.3710	0.0140	**9.51E-149**	no MDD	-0.3711	0.0051	**<5e-324**
trouble sleeping during worst anx.	RMDD	-0.0500	0.0145	0.000582775	SMDD	-0.0963	0.0289	0.00086212	no MDD	-0.1166	0.0106	**3.51E-28**
keyed up/on edge during worst anx.	RMDD	-0.0890	0.0137	**8.32E-11**	SMDD	-0.1290	0.0273	**2.29E-06**	no MDD	-0.1267	0.0102	**1.91E-35**
irratable during worst anx.	RMDD	-0.1294	0.0122	**3.19E-26**	SMDD	-0.1671	0.0246	**1.33E-11**	no MDD	-0.1830	0.0098	**1.92E-76**
multiple worries during worst anx.	RMDD	-0.2675	0.0131	**1.34E-91**	SMDD	-0.3405	0.0240	**1.04E-44**	no MDD	-0.2965	0.0096	**1.30E-205**
stronger worrying than other people during worst anx.	RMDD	-0.2350	0.0172	**4.78E-42**	SMDD	-0.2919	0.0335	**4.23E-18**	no MDD	-0.2055	0.0126	**4.84E-59**
tense/sore/aching muscles during worst anx.	RMDD	-0.1929	0.0103	**2.49E-77**	SMDD	-0.3255	0.0220	**2.84E-48**	no MDD	-0.2503	0.0101	**2.06E-132**

worried most days during worst anx.	RMDD	-0.1588	0.0194	**3.21E-16**	SMDD	-0.2219	0.0359	**7.06E-10**	no MDD	-0.1676	0.0118	**3.17E-45**
impact on normal roles during worst anx.	RMDD	-0.0896	0.0124	**4.48E-13**	SMDD	-0.1331	0.0237	**1.97E-08**	no MDD	-0.1359	0.0090	**2.58E-51**

C

**variable**	**group**	**beta**	**SE**	**p**	**group**	**beta**	**SE**	**p**	**group**	**beta**	**SE**	**p**
neuroticism score	RMDD	-0.4907	0.0060	**<5e-324**	SMDD	-0.5368	0.0091	**<5e-324**	no MDD	-0.4693	0.0028	**<5e-324**
fed up feelings	RMDD	-0.4352	0.0057	**<5e-324**	SMDD	-0.4858	0.0086	**<5e-324**	no MDD	-0.3962	0.0026	**<5e-324**
loneliness	RMDD	-0.4421	0.0062	**<5e-324**	SMDD	-0.5255	0.0103	**<5e-324**	no MDD	-0.4680	0.0036	**<5e-324**
miserableness	RMDD	-0.3450	0.0061	**<5e-324**	SMDD	-0.3832	0.0087	**<5e-324**	no MDD	-0.3282	0.0026	**<5e-324**
irritability	RMDD	-0.2793	0.0061	**<5e-324**	SMDD	-0.3281	0.0097	**2.59E-246**	no MDD	-0.2802	0.0029	**<5e-324**
sensitivity - hurt feelings	RMDD	-0.2100	0.0064	**7.92E-232**	SMDD	-0.2330	0.0093	**5.28E-137**	no MDD	-0.1916	0.0025	**<5e-324**
guilty feelings	RMDD	-0.2647	0.0059	**<5e-324**	SMDD	-0.2988	0.0094	**9.77E-217**	no MDD	-0.2572	0.0030	**<5e-324**
suffer from nerves	RMDD	-0.2460	0.0063	**<5e-324**	SMDD	-0.3301	0.0104	**1.36E-215**	no MDD	-0.2378	0.0035	**<5e-324**
tense or highly-strung	RMDD	-0.2854	0.0065	**<5e-324**	SMDD	-0.3516	0.0110	**5.40E-218**	no MDD	-0.3060	0.0038	**<5e-324**
worry too long after embarrassment	RMDD	-0.2110	0.0061	**2.69E-258**	SMDD	-0.2468	0.0090	**1.49E-161**	no MDD	-0.2108	0.0026	**<5e-324**
worrier - anxious feelings	RMDD	-0.2377	0.0065	**9.25E-287**	SMDD	-0.2804	0.0093	**1.85E-196**	no MDD	-0.2305	0.0025	**<5e-324**
nervous feelings	RMDD	-0.2263	0.0063	**5.90E-282**	SMDD	-0.2938	0.0100	**3.99E-184**	no MDD	-0.2495	0.0032	**<5e-324**
mood swings	RMDD	-0.3605	0.0060	**<5e-324**	SMDD	-0.4104	0.0087	**<5e-324**	no MDD	-0.3261	0.0026	**<5e-324**
risk taking	RMDD	0.0130	0.0067	0.050743831	SMDD	0.0310	0.0105	0.00325419	no MDD	0.0269	0.0029	**3.72E-20**

D

**variable**	**group**	**beta**	**SE**	**p**	**group**	**beta**	**SE**	**p**	**group**	**beta**	**SE**	**p**
days/week moderate physical activity	RMDD	0.1216	0.0060	**1.95E-90**	SMDD	0.1422	0.0091	**2.31E-54**	no MDD	0.0910	0.0026	**2.44E-264**
BMI	RMDD	-0.1572	0.0056	**1.19E-174**	SMDD	-0.1502	0.0085	**1.22E-69**	no MDD	-0.1179	0.0027	**<5e-324**
COPD diagnosed by doctor	RMDD	-0.2523	0.0229	**2.63E-28**	SMDD	-0.3645	0.0340	**8.63E-27**	no MDD	-0.2514	0.0125	**6.52E-90**
cardiovascular problems diagnosed by doctor	RMDD	-0.2249	0.0138	**8.26E-60**	SMDD	-0.2277	0.0196	**4.73E-31**	no MDD	-0.1891	0.0060	**3.32E-214**

diabetes diagnosed by doctor	RMDD	-0.3045	0.0139	**2.28E-106**	SMDD	-0.2581	0.0204	**1.12E-36**	no MDD	-0.2125	0.0060	**2.02E-271**
longstanding illness - disability - infirmity	RMDD	-0.3074	0.0060	**<5e-324**	SMDD	-0.3255	0.0094	**3.59E-257**	no MDD	-0.2610	0.0028	**<5e-324**
cancer diagnosed by doctor	RMDD	-0.0584	0.0109	**8.60E-08**	SMDD	-0.0615	0.0165	**0.00019557**	no MDD	-0.0698	0.0049	**5.30E-46**
used cannabis > 10 times	RMDD	-0.0818	0.0133	**8.69E-10**	SMDD	-0.1354	0.0242	**2.16E-08**	no MDD	-0.1402	0.0076	**1.11E-75**
typically 3-4 alcohol units vs max 2	RMDD	0.0073	0.0094	0.438561593	SMDD	0.0159	0.0153	0.30039099	no MDD	0.0085	0.0043	0.04909638
typically 5-6 alcohol units vs max 2	RMDD	-0.0224	0.0130	0.083671445	SMDD	-0.0110	0.0213	0.60440356	no MDD	-0.0076	0.0059	0.1973796
typically 7-9 alcohol units vs max 2	RMDD	-0.1104	0.0166	**2.68E-11**	SMDD	-0.0651	0.0288	0.02401163	no MDD	-0.0472	0.0078	**1.66E-09**
typically 10 alcohol units vs max 2	RMDD	-0.2131	0.0246	**5.78E-18**	SMDD	-0.0813	0.0411	0.04800248	no MDD	-0.0821	0.0118	**2.97E-12**
alcohol intake never/spec occasions vs 1-2/week	RMDD	-0.1300	0.0092	**1.57E-45**	SMDD	-0.1409	0.0140	**1.08E-23**	no MDD	-0.0845	0.0041	**8.24E-95**
alcohol intake 1-3/month vs 1-2/week	RMDD	-0.0448	0.0099	**6.18E-06**	SMDD	-0.0679	0.0151	**7.31E-06**	no MDD	-0.0524	0.0046	**2.39E-30**
alcohol intake 3-4/week vs 1-2/week	RMDD	0.0298	0.0084	0.000369909	SMDD	0.0380	0.0125	0.00233892	no MDD	0.0227	0.0035	**5.41E-11**
alcohol intake daily vs 1-2/week	RMDD	-0.0183	0.0087	0.035685672	SMDD	-0.0182	0.0132	0.16855169	no MDD	-0.0083	0.0037	0.02325069
six or more alcohol units < monthly vs never	RMDD	-0.0023	0.0099	0.815675971	SMDD	0.0194	0.0160	0.22550399	no MDD	-0.0041	0.0046	0.37677958
six or more alcohol units monthly vs never	RMDD	0.0035	0.0151	0.817749698	SMDD	0.0220	0.0247	0.37426042	no MDD	0.0402	0.0067	**2.32E-09**
six or more alcohol units weekly vs never	RMDD	-0.0400	0.0130	0.002099111	SMDD	-0.0108	0.0214	0.61394659	no MDD	-0.0091	0.0058	0.12140314
six or more alcohol units daily vs never	RMDD	-0.2214	0.0215	**7.99E-25**	SMDD	-0.1181	0.0382	0.00201153	no MDD	-0.1255	0.0108	**2.05E-31**

E

**variable**	**group**	**beta**	**SE**	**p**	**group**	**beta**	**SE**	**p**	**group**	**beta**	**SE**	**p**
MDD PRS	RMDD	-0.0630	0.0066	**1.91e-21**	SMDD	-0.0690	0.0102	**1.70e-11**	no MDD	-0.0575	0.0029	**8.79e-88**
Schizophrenia PRS	RMDD	-0.0399	0.0067	**2.26e-9**	SMDD	-0.0480	0.0102	**2.44e-6**	no MDD	-0.0412	0.0029	**1.14e-45**
Bipolar disorder PRS	RMDD	-0.0138	0.0067	0.0385	SMDD	-0.0162	0.0103	0.1159	no MDD	-0.0129	0.0029	**1.08e-5**

Anxiety PRS	RMDD	-0.0208	0.0066	**1.56e-3**	SMDD	-0.0292	0.0101	**3.69e-3**	no MDD	-0.0207	0.0029	**5.97e-13**
Alcohol dependence PRS	RMDD	-0.0240	0.0068	**4.15e-4**	SMDD	-0.0255	0.0104	0.01446	no MDD	-0.0145	0.0030	**1.15e-6**
ADHD PRS	RMDD	-0.0310	0.0066	**2.53e-6**	SMDD	-0.0377	0.0103	**2.26e-4**	no MDD	-0.0128	0.0029	**8.31e-6**
Neuroticism PRS	RMDD	-0.0415	0.0066	**3.24e-10**	SMDD	-0.0380	0.0101	**1.75e-4**	no MDD	-0.0297	0.0029	**4.11e-25**

Supplementary Table 6: power estimation for PRS analysis using the R library 'avengeme'. We assumed that 0.95 of SNPs had no effect on the training trait and considered a Bonferroni corrected p threshold of 2.4e-3. Genetic covariance was calculated as a function of SNP-heritability (SNP-h2) of the traits of interest and genetic correlation with wellbeing reported in the base genome-wide association studies (GWASs). For references to the base GWASs see Supplementary Table 1. MDD=major depressive disorder; SCZ=schizophrenia; BP=bipolar disorder; ADHD=attention-deficit hyperactivity disorder.

Phenotype	Base GWAS N	SNP-h2 of base phenotype	Genetic covariance	Group in target data	Power
MDD	143,265	0.09	0.094	RMDD	0.99
				SMDD	0.84
				No MDD	1
SCZ	105,318	0.33	0.076	RMDD	0.99
				SMDD	0.83
				No MDD	1
BP	51,710	0.35	0.050	RMDD	0.43
				SMDD	0.12
				No MDD	0.99
ADHD	55,374	0.24	0.053	RMDD	0.30
				SMDD	0.08
				No MDD	0.98
Anxiety disorders	21,761	0.14	0.087	RMDD	0.22
				SMDD	0.06
				No MDD	0.94
Alcohol dependence	52,848	0.09	0.057	RMDD	0.29
				SMDD	0.08
				No MDD	0.98
Neuroticism	63,030	0.15	0.113	RMDD	0.99
				SMDD	0.70
				No MDD	1

Supplementary Table 7: combined analyses of the significant predictors in each considered category using elastic net regression; the functioning-wellbeing score (FWS) values predicted by the elastic net model were tested for prediction of the observed FWS values using linear regression to obtain the results showed in this table. In the column "N" we specified how many individuals were included in the analysis, as those with missing values in one or more of the considered predictors had to be excluded. R^2 represents the variance in FWS explained by the model. RMDD=recurrent major depressive disorder; SMDD=single episode major depressive disorder. PRSs=polygenic risk scores.

**Predictor category**	**Group**	**N**	**beta**	**SE**	**P**	**R^2**
Depressive symptoms/characteristics	RMDD	6,559	0.4376	0.0112	2.45e-301	0.2012
	SMDD	1,713	0.4366	0.0219	7.93e-80	0.1959
	No MDD	4,252	0.4312	0.0140	3.61e-187	0.1915
Anxiety symptoms/characteristics	RMDD	11,205	0.2424	0.0092	6.64e-148	0.0634
	SMDD	2,791	0.3176	0.0181	2.76e-65	0.1028
	No MDD	9,504	0.3283	0.0098	8.04e-233	0.1089
Neuroticism-related personality traits	RMDD	39,460	0.4255	0.0045	p<5e-324	0.1918
	SMDD	16,909	0.4561	0.0068	p<5e-324	0.2165
	No MDD	165,654	0.4060	0.0022	p<5e-324	0.1718
Depressive symptoms/characteristics + neuroticism	RMDD	5,805	0.5164	0.0113	p<5e-324	0.2752
	SMDD	1,486	0.5250	0.0221	1.02e-105	0.2811
	No MDD	3,764	0.5238	0.0140	7.27e-260	0.2790
Lifestyle and physical diseases	RMDD	25,283	0.2240	0.0061	9.66e-287	0.0543
	SMDD	10,076	0.2232	0.0097	2.88e-114	0.0530
	No MDD	95,483	0.1952	0.0032	p<5e-324	0.0405
PRSs	RMDD	38,530	0.0615	0.0051	1.73e-33	0.0046
	SMDD	16,503	0.0671	0.0078	7.55e-18	0.0055
	No MDD	158,041	0.0610	0.0025	6.46e-129	0.0048

Supplementary Table 8: results of the mediation analysis, using neuroticism as mediator of the depressive symptoms/characteristics associated with FWS in at least two groups of individuals. Depression is referred as MDD or dep. The predictors in bold showed heterogeneous effects among the considered groups of individuals.

		Recurrent MDD			Single episode MDD			No MDD		
Mediator	Predictor	Direct effect	Indirect effect	Prop. mediated	Direct effect	Indirect effect	Prop. mediated	Direct effect	Indirect effect	Prop. mediated
Neuroticism	**Ever self-harm**	-0.251 (-0.279/-0.226)	-0.059 (-0.069/-0.049)	0.190 **(**0.159/0.224)	-0.265 (-0.318/-0.210)	-0.109 (-0.134/-0.085)	0.294 (0.234/0.357)	-0.320 (-0.345/-0.290)	-0.105 (-0.117/-0.093)	0.246 (0.221/0.274)
	**Ever contemplated self-harm**	-0.336 (-0.352/-0.320)	-0.058 (-0.065/-0.053)	0.148 (0.134/0.164)	-0.383 (-0.417/-0.352)	-0.091 (-0.104/-0.077)	0.191 (0.164/0.219)	-0.430 (-0.445/-0.414)	-0.104 (-0.110/-0.099)	0.195 (0.185/0.206)
	Death thoughts during dep.	-0.229 (-0.245/-0.213)	-0.052 (-0.058/-0.046)	0.186 (0.165/0.205)	-0.224 (-0.251/-0.196)	-0.052 (-0.063/-0.041)	0.187 (0.150/0.228)	-0.144 (-0.157/-0.131)	-0.042 (-0.047/-0.037)	0.225 (0.199/0.251)
	Worthlessness during dep.	-0.239 (-0.256/-0.222)	-0.101 (-0.108/-0.094)	0.297 (0.0277/0.320)	-0.201 (-0.227/-0.172)	-0.124 (-0.136/-0.111)	0.382 (0.342/0.427)	-0.281 (-0.300/-0.266)	-0.117 (-0.123/-0.111)	0.294 (0.277/0.311)
	Prolonged loss of interest during dep.	-0.199 (-0.221/-0.180)	-0.059 (-0.066/-0.051)	0.227 (0.198/0.257)	-0.156 (-0.182/-0.131)	-0.58 (-0.070/-0.047)	0.271 (0.220/0.329)	-0.248 (-0.258/-0.238)	-0.094 (-0.098/-0.090)	0.275 (0.262/0.287)
	**Impact on normal roles during dep.**	-0.098 (-0.124/-0.072)	-0.008 (-0.018/0.001)	0.072 **(**-0.013/0.157)	-0.057 (-0.107/-0.008)	-0.036 (-0.055/-0.016)	0.390 (0.181/0.785)	-0.066 (-0.079/-0.054)	-0.020 (-0.026/-0.016)	0.236 (0.185/0.292)
	Weight gain during dep.	-0.143 (-0.165/-0.117)	-0.044 (-0.053/-0.035)	0.235 (0.190/0.289)	-0.128 (-0.175/-0.082)	-0.068 (-0.089/-0.048)	0.346 (0.253/0.470)	-0.196 (-0.218/-0.176)	-0.066 (-0.074/-0.057)	0.252 (0.219/0.284)
	Weight loss during dep.	0.029 (0.010/0.047)	-0.004 (-0.012/0.003)	-0.157 (-1.095/0.140)	0.064 (0.031/0.098)	0.004 (-0.01/0.018)	0.057 (-0.223/0.245)	0.032 (0.017/0.048)	-0.004 (-0.009/-0.002)	-0.113 (-0528/0.075)
	Both weight gain and loss during dep.	-0.134 (-0.170/-0.098)	-0.041 (-0.053/-0.029)	0.235 (0.167/0.313)	-0.118 (-0.189/-0.052)	-0.079 (-0.110/-0.045)	0.400 (0.248/0.629)	-0.135 (-0.169/-0.103)	-0.067 (-0.080/-0.055)	0.332 (0.270/0.404)
	Sleeping too much during dep.	-0.113 (-0.135/-0.092)	-0.033 (-0.040/-0.026)	0.225 (0.178/0.284)	-0.119 (-0.158/-0.082)	-0.045 (-0.060/-0.028)	0.273 (0.179/0.378)	-0.147 (-0.172/-0.122)	-0.043 (-0.053/-0.034)	0.228 (0.183/0.279)
	Tiredness during dep.	-0.057 (-0.088/-0.024)	-0.041 (-0.053/-0.030)	0.419 (0.296/0.627)	-0.033 (-0.079/0.012)	-0.054 (-0.074/-0.035)	0.625 (0.381/1.295)	-0.101 (-0.115/-0.087)	-0.061 (-0.066/-0.056)	0.378 (0.340/0.419)
	Concentration difficulties during dep.	-0.044 (-0.073/-0.015)	-0.041 (-0.051/-0.030)	0.485 (0.333/0.728)	-0.053 (-0.099/-0.006)	-0.044 (-0.063/-0.024)	0.449 (0.259/0.876)	-0.089 (-0.104/-0.075)	-0.058 (-0.064/-0.053)	0.395 (0.353/0.443)
	**Ever unenthusiastic for one whole week**	-0.121 (-0.136/-0.106)	-0.096 (-0.104/-0.089)	0.442 (0.409/0.480)	-0.106 (-0.126/-0.086)	-0.072 (-0.083/-0.062)	0.404 (0.348/0.470)	-0.109 (-0.133/-0.086)	-0.054 (-0.064/-0.044)	0.332 (0.272/0.402)

	**Dep. possibly related to traumatic event**	0.063 (0.046/0.083)	0.039 (0.032/0.045)	0.381 (0.305/0.468)	0.075 (0.044/0.105)	0.054 (0.040/0.068)	0.419 (0.312/0.568)	0.142 (0.127/0.157)	0.045 (0.039/0.050)	0.240 (0.211/0.270)
	Chronic dep.	-0.191 (-0.217/-0.164)	-0.058 (-0.067/-0.049)	0.232 (0.197/0.270)	-0.356 (-0.420/-0.288)	-0.154 (-0.179/-0129)	0.303 (0.253/0.359)	-0.247 (-0.277/-0.217)	-0.078 (-0.089/-0.068)	0.241 (0.208/0.277)

Supplementary Figure 1: distribution of the scores used to create the FWS. For measures taken at different time points, all the time points are reported with the respective numerosity. Note that all variables are between 1 and 6, except belief that own life is meaningful (1-5) and that own life is worth living (0-2), and that scores were harmonized to make higher scores correspond to better wellbeing.

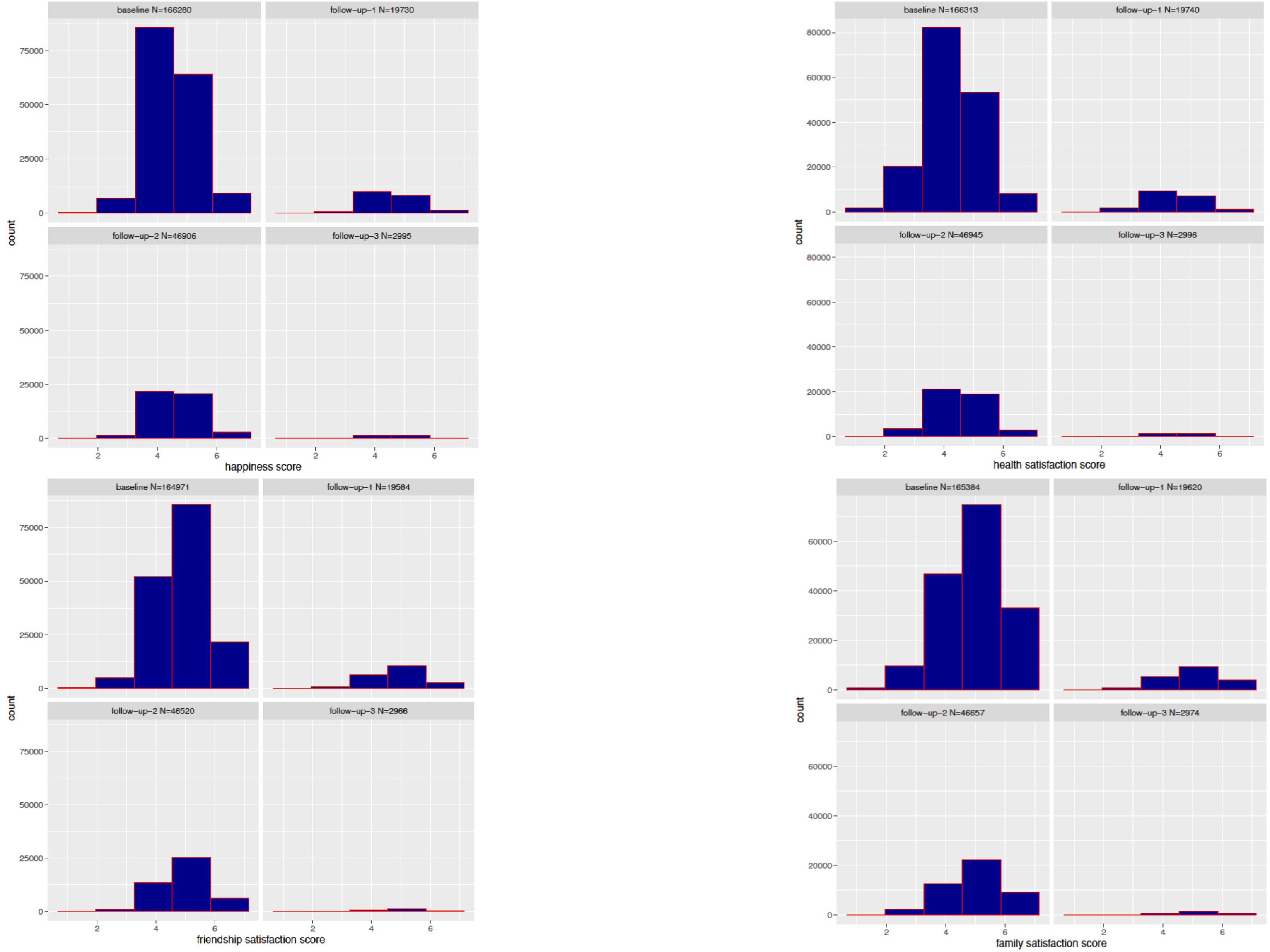

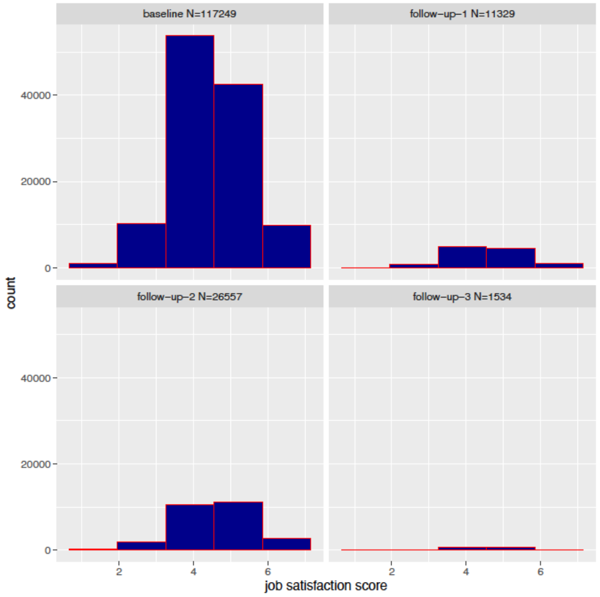
baseline N=117249
follow−up−1 N=11329
follow−up−2 N=26557
follow−up−3 N=1534
count
job satisfaction score

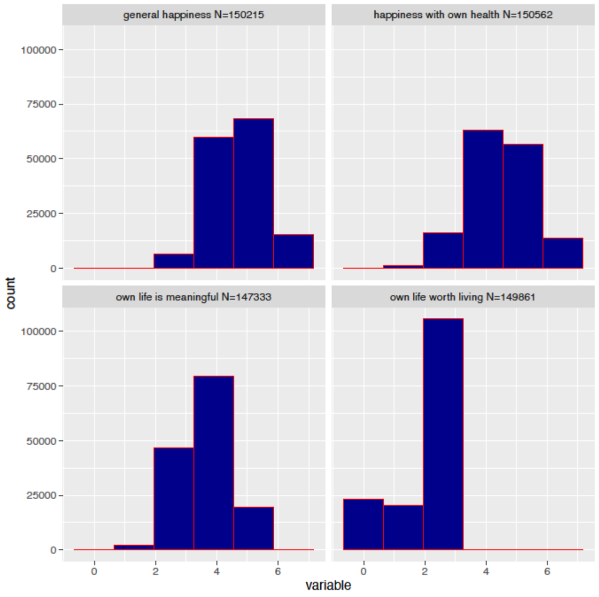
general happiness N=150215
happiness with own health N=150562
own life is meaningful N=147333
own life worth living N=149861
count
variable

Supplementary Figure 2: selection of the number factors to capture the four components of the functional-wellbeing score (FWS). The optimal coordinates (OC) and acceleration factor (AF) techniques are used to identify an elbow in the scree plot. The Kaiser rule and parallele analysis were also used (the four factors simulated in parallel analysis have Eigenvalues very close to 1 which small differences are not visibile).

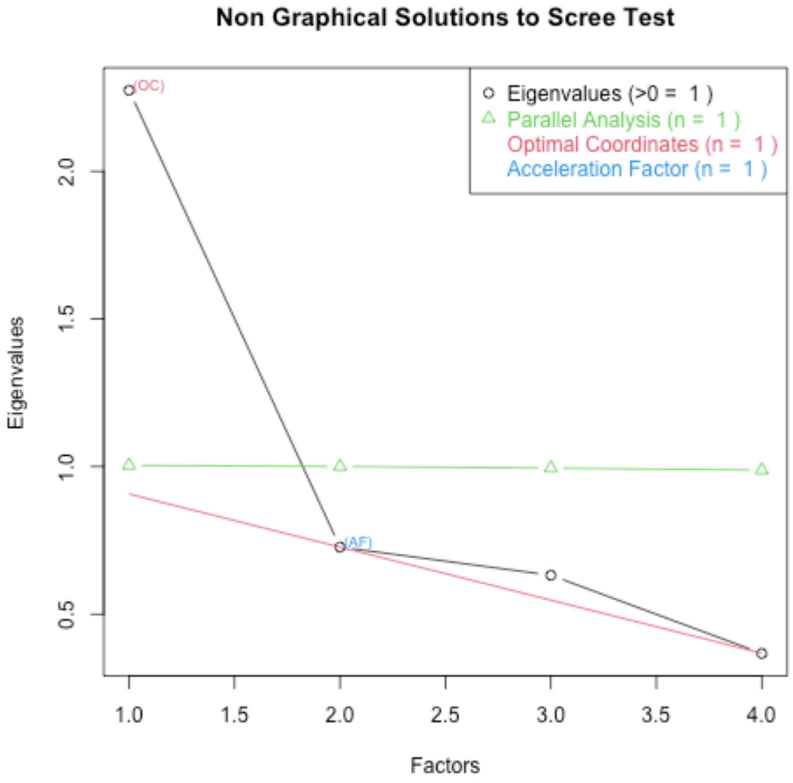

Supplementary Figure 3: distribution of the functioning-wellbeing score (FWS) in the considered groups of individuals, absolute counts in **A** and density plot in **B**.

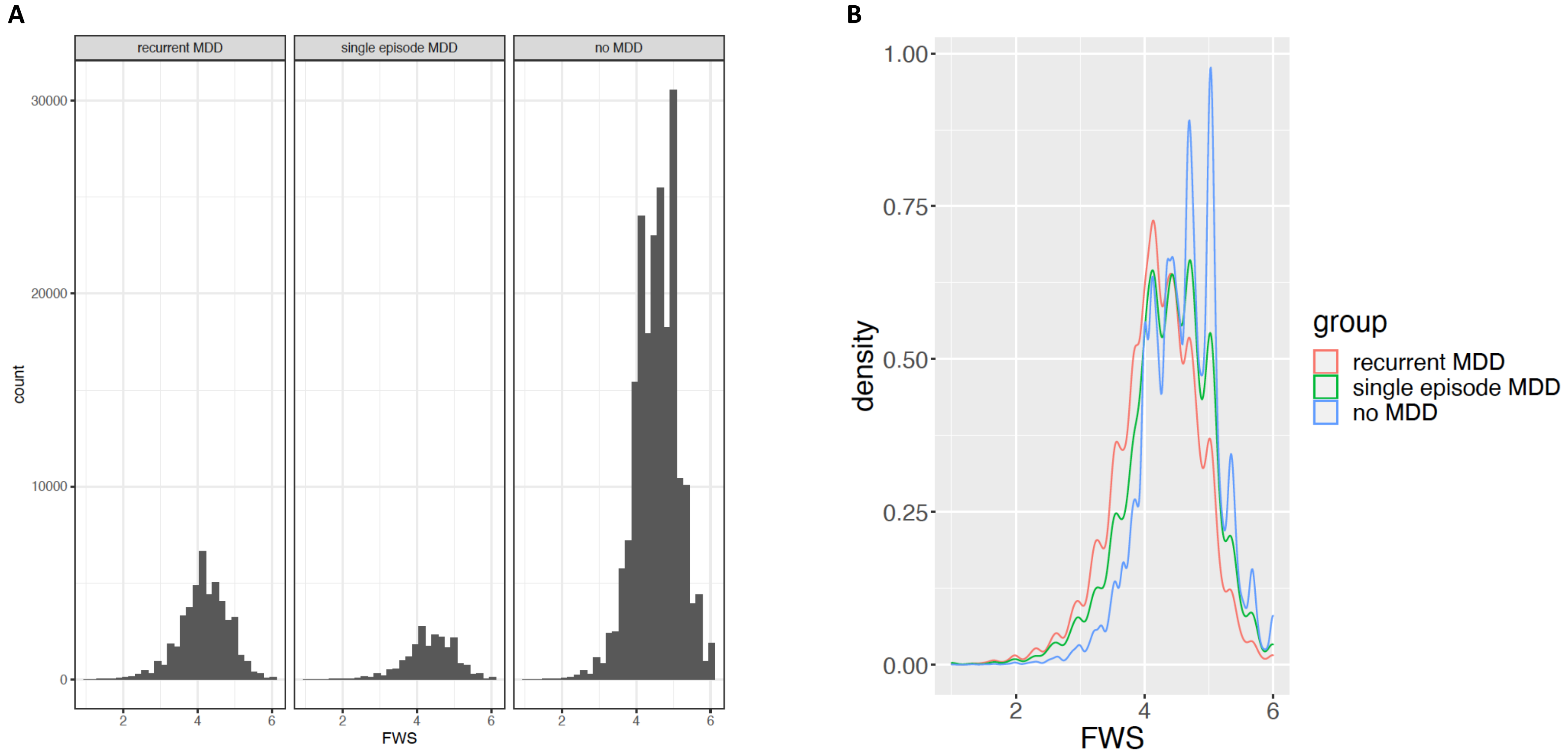

Supplement: Supplementary file 1 [file S003329172100502Xsup001.pdf]
